# Nonlinear viscoelastic constitutive model for bovine liver tissue

**DOI:** 10.1007/s10237-020-01297-5

**Published:** 2020-02-10

**Authors:** Adela Capilnasiu, Lynne Bilston, Ralph Sinkus, David Nordsletten

**Affiliations:** 1 Division of Biomedical Engineering and Imaging Sciences, King’s College London, London, UK; 2 Inserm U1148, LVTS, University Paris Diderot, University Paris 13, 75018 Paris, France; 3 Department of Biomedical Engineering and Cardiac Surgery, University of Michigan, Ann Arbor, USA; 4 Prince of Wales Clinical School, University of New South Wales, Sydney, Australia; 5 Neuroscience Research Australia, Sydney, Australia

**Keywords:** Liver rheology, Biomechanics, Nonlinear mechanics, Viscoelasticity

## Abstract

Soft tissue mechanical characterisation is important in many areas of medical research. Examples span from surgery training, device design and testing, sudden injury and disease diagnosis. The liver is of particular interest, as it is the most commonly injured organ in frontal and side motor vehicle crashes, and also assessed for inflammation and fibrosis in chronic liver diseases. Hence, an extensive rheological characterisation of liver tissue would contribute to advancements in these areas, which are dependent upon underlying biomechanical models. The aim of this paper is to define a liver constitutive equation that is able to characterise the nonlinear viscoelastic behaviour of liver tissue under a range of deformations and frequencies. The tissue response to large amplitude oscillatory shear (1–50%) under varying preloads (1–20%) and frequencies (0.5–2 Hz) is modelled using viscoelastic-adapted forms of the Mooney–Rivlin, Ogden and exponential models. These models are fit to the data using classical or modified objective norms. The results show that all three models are suitable for capturing the initial nonlinear regime, with the latter two being capable of capturing, simultaneously, the whole deformation range tested. The work presented here provides a comprehensive analysis across several material models and norms, leading to an identifiable constitutive equation that describes the nonlinear viscoelastic behaviour of the liver.

## Introduction

Biomechanical characterisation of tissues is essential in medical research. New surgery techniques, implants or devices are being tested in silico, in vitro and in vivo (O’Toole et al. 1995; Marescaux et al. 1998; Rosen et al. 2008; Clin et al. 2010; Gonzalez-Blohm et al. 2015). In these tests, it is critical to know the liver’s response to a range of factors, such as puncturing, cutting, deformations and displacements. For diagnosis purposes, elastography is a technique that depends on the underlying tissue properties in order to assess the presence of disease (Fovargue et al. 2018). In vivo magnetic resonance elastography (MRE) has shown that the existence of liver inflammation and fibrosis gives higher stiffness measurements (Huwart et al. 2006; Sinkus et al. 2018). However, coexisting diseases (Mueller et al. 2010) and the bias introduced by large deformations on elastography measurements (Capilnasiu et al. 2019) can further complicate the diagnosis. Comprehensive liver models could also benefit other research areas like transportation safety (Viano et al. 1989; Yoganandan et al. 2000), where biomechanical tests for abdominal injury tolerance limits provide essential information in developing safer vehicles (Kemper et al. 2010). With the liver being the most frequently injured organ in frontal and side impacts (Yoganandan et al. 2000), a finite element (FE) model could be used for prediction purposes, provided that it incorporates local and global liver tissue response to mechanical testing. Hence, improved knowledge of the nonlinear viscoelastic behaviour of the liver is needed.

Over the past decades, a range of rheological tests have been employed to characterise liver tissue, the most common being uniaxial deformation (either as small sample loading or indentation on the full organ) and shearing. Both oscillatory shear and uniaxial deformation tests show that, at low strains, the liver exhibits quasi-linearity, with the nonlinear behaviour being exposed at higher strains (Liu and Bilston 2000; Gao et al. 2010; Tan et al. 2013). Additionally, loading–unloading tests reveal that hysteresis effects are taking place (Jordan et al. 2011), with the response being rate dependent (Liu and Bilston 2000; Miller 2000). Multi-frequency soft tissue measurements of the shear modulus $G^*$ indicate a fractional-order dependence on the angular frequency in the form of $G^* \propto \omega ^\alpha$ (Holm and Sinkus 2010), with $\alpha \in [0.2, 0.35]$ [e.g. $\alpha \approx 0.23$ (Liu and Bilston 2000), $\alpha \approx 0.26$ (Jordan et al. 2011; Sinkus et al. 2018), $\alpha \approx 0.32$ (Asbach et al. 2008)]. Other biomechanical properties of the liver have also been investigated, such as relaxation (Liu and Bilston 2006; Chatelin et al. 2011) and creep (Wang et al. 1992).

Liver tissue rheology measurements have lead to a range of biomechanical models. Hyperelasticity is often assumed, with polynomial, exponential and logarithmic forms being employed for compression and elongation data (Chui et al. 2004; Gao et al. 2010). The general findings indicate that the exponential, logarithmic and power law models offer more flexibility in capturing the different regions of the stress–strain curves. In order to probe viscoelasticity, cyclic deformations or relaxation tests usually need to be investigated. Some studies employed relaxation (Liu and Bilston 2006), shear oscillations (Nicolle and Palierne 2015) or cyclic indentation (Jordan et al. 2011) over a range of frequencies, thus offering a broader picture of the biomechanical behaviour of liver. Among these, the K-BKZ model was proposed due to its awareness of the complete past time history and was validated against small amplitude oscillatory shear and strain ramp (Nicolle and Palierne 2015). Alternatively, viscoelasticity was modelled by introducing a Maxwell element. A complex differential model, with ten model parameters, was investigated by Liu and Bilston (2006) against the relaxation behaviour at four strain levels. Ayyildiz et al. (2015) also proposed a Maxwell-based model with 13 parameters for capturing the viscoelastic behaviour of liver at a range of uniaxial preloads, frequencies and strain rates. There, large preloads (20%) and shear strains (5%) were employed simultaneously in the testing protocol. However, the results focus on the effect of preload, strain rate and frequency on the normal force and torque response, while the combined effect of large preloads and shear strains is not addressed. Jordan et al. (2011) considered increasingly complex networks of springs and dashpots, arranged both in series and in parallel, in order to model the liver behaviour under cyclic indentation at different strain rates and relaxation. A power law model considering solid-phase compressibility was employed by Perepelyuk et al. (2016), who, at large preload strains and small oscillatory shear, measured the storage modulus $G^\prime$. While these models significantly contribute to our understanding of separate aspects of the viscoelastic behaviour of the liver, there remains a need for a comprehensive 3D model which can describe the tissue response under various deformation types and frequencies.

In this paper, we present a comparison of liver constitutive models based on the tissue’s response to a range of large deformations and frequencies. A cross testing of uniaxial preloads (1–20%), shear strains (1–50%) and frequencies (0.5–2 Hz) is considered (Tan et al. 2013), thus emphasising the rate-dependent, nonlinearly viscoelastic behaviour of the liver. The testing protocol displays a strain softening effect, which is addressed by proposing a new error norm that allows for some degree of flexibility in fitting the linear parameters of the models. This analysis and model-fitting procedure lead to the identification of simplified constitutive models, which retain the essential components needed for characterising the above-mentioned properties of the liver exhibited under combined deformation and various frequencies. To the authors’ knowledge, this is one of the first liver studies that investigates combined large uniaxial and shear loading, at various frequencies, and the first study that proposes a three-dimensional nonlinear viscoelastic model which can capture the large amplitude oscillatory response across a range of preloads and frequencies.

In what follows, a brief introduction to kinematics (Sect. 2.1) precedes the outlining of the experimental design and modelling assumptions (Sect. 2.2). Three different constitutive models are proposed, which are then fit to the data using a set of error norms that infer different model properties. Throughout Sect. 3, the results of the model-fitting process are going to be presented grouped by the norm investigated (Sects. 3.1–3.3), with the three models being compared within each subsection. This is followed by a discussion reviewing the findings and potential future applications (Sect. 4).

## Materials and methods

The aim of this work is to characterise the constitutive behaviour of the liver under a range of combined deformations and frequencies. In order to achieve this, Sect. 2.1 outlines the kinematics metrics that are needed throughout this paper (see Taber 2004; Bonet and Wood 2008). Section 2.2 explains the testing protocol and its modelling characteristics. Three different types of models are proposed in Sect. 2.3, which are due to be fit to the data using the methods described in Sect. 2.4.

### Kinematics background

Let the region $\varOmega _0\subset {\mathbb {R}}^{3}$ define a solid body which can be deformed in space and time using a displacement field $\varvec{U}: \varOmega _0\times [0, T] \rightarrow {\mathbb {R}}^3$. A point in the reference domain, $\varvec{X} \in \varOmega _0$, corresponds at time $t \in [0, T]$ to a point in the physical domain $\varOmega _t$, by the mapping

1 $$\begin{aligned} \varvec{x} = \varvec{U}(\varvec{X},t) + \varvec{X}, \qquad \varvec{x} \in \varOmega _t . \end{aligned}$$

The deformation gradient tensor $\varvec{F}$ relates the reference and physical domains via

2 $$\begin{aligned} \varvec{F}=\nabla _{\varvec{X}} \varvec{x} = \dfrac{\partial \varvec{x}}{\partial \varvec{X}}, \qquad F_{ij} = \dfrac{\partial x_i}{\partial X_j} . \end{aligned}$$

The volumetric changes between the two states are quantified by the determinant $J = \det \varvec{F}$, with $J=1$ implying incompressibility. From the deformation gradient $\varvec{F}$, the right and left Cauchy Green strains are defined as

$$\begin{aligned} \varvec{C}= \varvec{F}^{\mathrm{T}} \varvec{F}, \quad \varvec{B}= \varvec{F}\varvec{F}^{\mathrm{T}}, \end{aligned}$$

or, in their isochoric form (Bonet and Wood 2008), here indicated by the “$\hat{\quad }$” symbol, as

$$\begin{aligned} \hat{\varvec{F}} = J^{-1/3} \varvec{F}, \quad \hat{\varvec{C}} = \hat{\varvec{F}}^{\mathrm{T}} \hat{\varvec{F}}, \quad \hat{\varvec{B}} = \hat{\varvec{F}}\hat{\varvec{F}}^{\mathrm{T}}. \end{aligned}$$

Some tensor quantities that remain unchanged under rotations are the first and second invariants (Bonet and Wood 2008), obtained using the double contraction “:”

3 $$\begin{aligned} I_{{\varvec{A}}} = {\varvec{A}}:{\varvec{I}}, \quad II_{{\varvec{A}}} = {\varvec{A}}:{\varvec{A}}, \end{aligned}$$

where ${\varvec{A}}$ is a general $m \times m$ tensor. For clarity, in index notation, this is equivalent to

4 $$\begin{aligned} I_{{\varvec{A}}} =\sum _{i=1}^m \sum _{j=1}^m A _{ij} \delta _{ij}, \quad II_{{\varvec{A}}} = \sum _{i=1}^m \sum _{j=1}^m A _{ij} A _{ij}. \end{aligned}$$

Constitutive equations can be used to describe a material’s behaviour under deformation. Let $W({\varvec{C}})$ denote a strain energy function which depends on strain metrics, here in particular on ${\varvec{C}}$. The corresponding second Piola–Kirchhoff (PK2) tensor is obtained by taking the derivative of the strain energy function with respect to ${\varvec{C}}$, as ${\varvec{S}} = 2\nabla _{{\varvec{C}}}W$ (Bonet and Wood 2008). For a viscoelastic material description, fractional-order viscoelastic models have been successfully employed in modelling soft tissue behaviour (Kiss et al. 2004). Thus, here let ${\varvec{S}}$ be defined as the sum of an elastic, fractional viscoelastic and hydrostatic part, as

5 $$\begin{aligned} {\varvec{S}} = {\varvec{S}}_{\mathrm{e}} + D_t^\alpha {\varvec{S}}_{\mathrm{v}} + {\varvec{S}}_{\mathrm{p}}. \end{aligned}$$

The elastic and viscoelastic parts are derivatives of elastic and viscoelastic strain energy functions (i.e. ${\varvec{S}}_{\mathrm{e}} = 2\nabla _{{\varvec{C}}}W_{\mathrm{e}}$ and ${\varvec{S}}_{\mathrm{v}} = 2\nabla _{{\varvec{C}}}W_{\mathrm{v}}$), and the hydrostatic part is defined as ${\varvec{S}}_{\mathrm{p}} = J P {\varvec{C}}^{-1}$, where *P* is the hydrostatic pressure. The fractional-order derivative, as defined by Caputo (1967), is

6 $$\begin{aligned} D_t^\alpha {\varvec{S}}_{\mathrm{v}} = \frac{1}{\varGamma (1-\alpha )} \int _0^t \frac{1}{(t-z)^\alpha }\partial _t{\varvec{S}}_{\mathrm{v}}(z) {\text { d}} z , \end{aligned}$$

with $\alpha =0$ leading to a hyperelastic contribution and $\alpha =1$ leading to a purely viscous contribution in the form $\partial _t{\varvec{S}}_{\mathrm{v}}$. In order to separate the deviatoric and hydrostatic stress components, we introduce the deviatoric operator

7 $$\begin{aligned} {\text {Dev}}[{\varvec{A}}] = {\varvec{A}} - \dfrac{{\varvec{A}}: {\varvec{C}}}{3} {\varvec{C}}^{-1}, \end{aligned}$$

which ensures that ${\text {Dev}}[{\varvec{A}}]:{\varvec{C}}=0$. Having defined the PK2 tensor in the context of constitutive modelling, the Cauchy stress tensor can be related to the PK2 tensor using

8 $$\begin{aligned} {\varvec{\sigma }} = \dfrac{1}{J}{\varvec{F}} {\varvec{S}} {\varvec{F}}^{\mathrm{T}}. \end{aligned}$$

### Nonlinear viscoelastic characterisation of liver tissue

In this paper, combined loading experiments are used to investigate the behaviour of liver tissue. The data presented here have been previously published in Tan et al. (2013). Here, we focus on the large amplitude oscillatory strain (LAOS) tests and briefly review the protocol.

#### Sample preparation

Fresh healthy bovine liver was collected from an abattoir, with the samples being tested within 6 hours post-mortem. During transport, the livers were wrapped in saline-soaked gauze and transported on ice in a sealed container. Cylindrical samples were cut to approximately 10 mm radius and 3 mm height. During testing, in order to ensure hydration, the samples were maintained in a 100% humidity chamber, which is a part of the rheometer. The temperature was controlled to be 25 °C. For more complete preparatory details, see the original protocol published in Tan et al. (2013).

#### Rheological experimental design

**Fig. 1:**
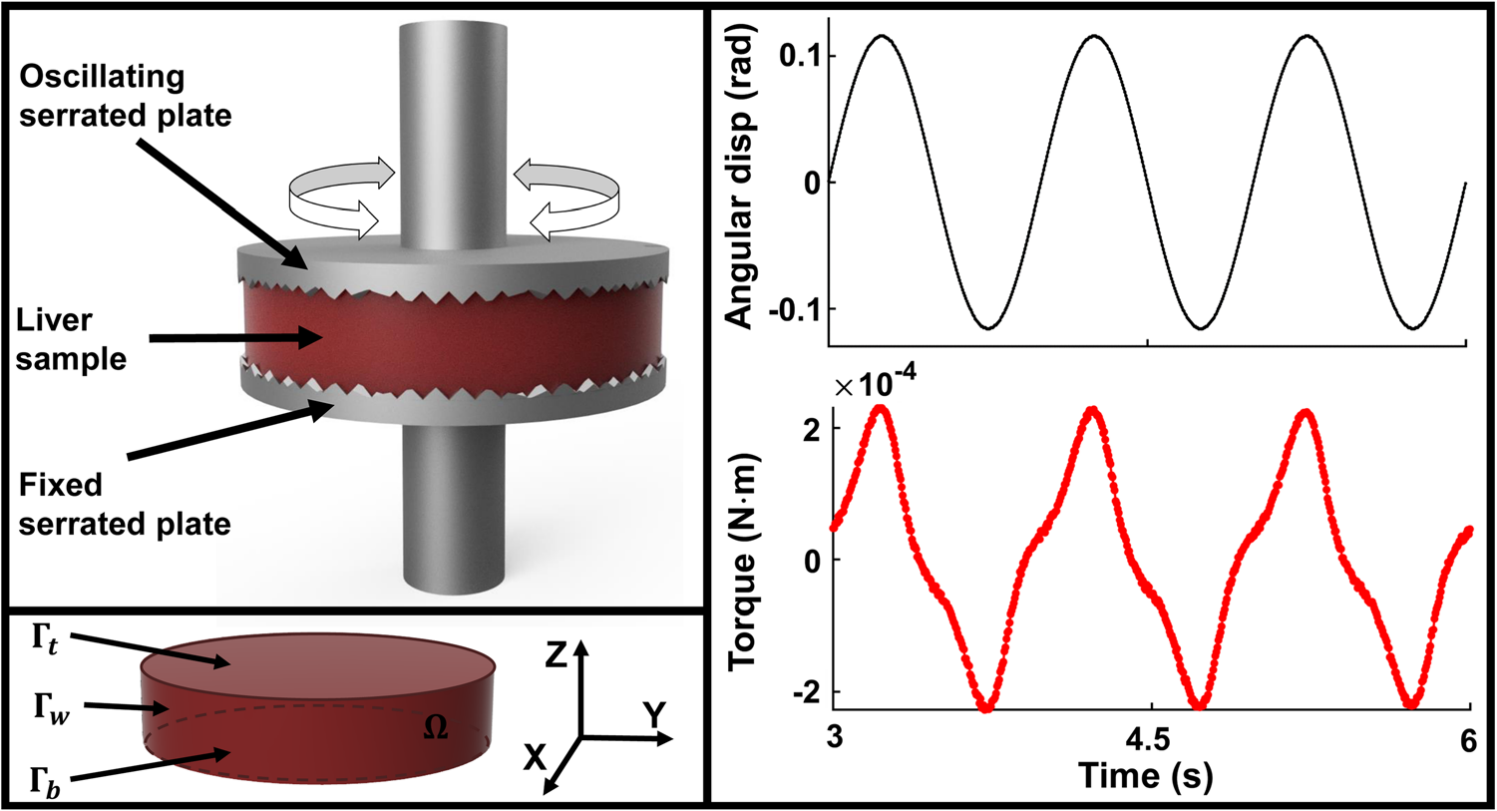
Illustration of the experimental setup. (Top left) Liver tissue in the oscillatory rheological instrument. The plates were serrated, with the lower plate being fixed, while the upper plate could move vertically and rotate around the axis. (Bottom left) Axis and boundaries defined with respect to the liver sample. (Right) Example of angular displacement trace (sinusoidal) and torque response (non-sinusoidal) at CS 10%, $\gamma$ 50%, 1 Hz

Tissues were tested using a rotational rheometer (Kinexus Pro KNX 2100, Malvern, United Kingdom), as illustrated in Fig. 1. The cylindrical samples were fit in between two serrated plates of 20 mm diameter, to avoid slipping. The bottom plate was fixed, while the upper plate was vertically adjusted and oscillated around the cylindrical axis. Torque measurements were acquired in combined loading tests, with shear strains of 1, 10, 25 and 50% being investigated under different uniaxial preload levels—1, 10 and 20%, at a strain rate of 1 Hz. Additionally, shear strains of 1, 10 and 25%, at a preload of 10%, were investigated at strain rates of 0.5 and 2 Hz. Table 1 summarises the loading, shearing and frequency protocols employed.

**Table 1:** Testing protocol across frequencies, compression and shear deformations

	SS 1%	SS 10%	SS 25%	SS 50%
CS 1%	${{\bigcirc }}$	${{\bigcirc }}$	${{\bigcirc }}$	${{\bigcirc }}$
CS 10%	$\bigoplus$	$\bigoplus$	$\bigoplus$	${{\bigcirc }}$
CS 20%	${{\bigcirc }}$	${{\bigcirc }}$	${{\bigcirc }}$	${{\bigcirc }}$
Legend	| 0.5 Hz	${{\bigcirc }}$ 1Hz	− 2Hz	

A total of 18 tests were carried out—12 at 1 Hz and 3 at 0.5 and 2 Hz, respectively. For each test, the data were averaged between at least four liver samples. For each test, the first eight cycles were used for preconditioning purposes, with the data being recorded from the ninth cycle. Preconditioning was carried out sequentially and not directly at the maximum deformation level in order to avoid damaging tissue (e.g. eight preconditioning cycles at shear strain 1% and then three data cycles, followed by eight preconditioning cycles at shear strain 10% and then three data cycles, etc). Originally, two more shear strain levels were acquired—80 and 100% (Tan et al. 2013), but these data were excluded here due to potential tissue damage.

#### Modelling the experiment

Compressive and shear deformations were imposed onto the liver samples in order to investigate their 3D biomechanical response. Thus, denoting by *H* and *R* the undeformed height and radius of a sample, let *h* and *r* denote the height and radius deformed by compression. Let the ratio of the deformed to undeformed height be $\lambda = {h} / {H}$, which corresponds to each compression strain (CS) level via ${\text {CS}}=1-\lambda$. Here, ideal compression is assumed, which leads to the radius being deformed as $r ={R} /{\sqrt{\lambda }}$.

The shear strain, $\gamma$, is defined as the ratio between top plate rotational part of the displacement and inter plate gap, $\gamma ={d} / {h}$, with the rotational displacement depending on the angular displacement and radius. Having a predefined $\gamma$ level, at frequency *f* the angular displacement on the top of the sample is given by

9 $$\begin{aligned} \psi (t)=\dfrac{\sin (2\pi f t) h \gamma }{r}, \end{aligned}$$

with the maximum angular displacement being reached at $\psi = { h \gamma }/{r}$. Let $\varPsi (t,X_3)$ define the angular displacement throughout the sample, as

$$\begin{aligned} \varPsi (t,X_3) = \dfrac{\psi (t)}{H} X_3. \end{aligned}$$

Hence, the compression and shearing lead to a body deformation (Taber 2004) defined by

10 $$\begin{aligned} {\varvec{x}} (t)= \left[ \begin{array}{c} \dfrac{X_1 }{\sqrt{\lambda }} \cos \left( \varPsi (t,X_3) \right) -\dfrac{ X_2 }{\sqrt{\lambda }} \sin \left( \varPsi (t,X_3) \right) \\ \dfrac{X_1}{\sqrt{\lambda }} \sin \left( \varPsi (t,X_3) \right) + \dfrac{ X_2 }{\sqrt{\lambda }} \cos \left( \varPsi (t,X_3) \right) \\ \lambda X_3 \\ \end{array} \right] . \end{aligned}$$

Incorporating the above form into Eq. 2, the corresponding deformation gradient takes the form

11 $$\begin{aligned}{\varvec{F}}(t) =\left( \begin{array}{ccc} \dfrac{\cos \varPsi (t,X_{3})}{\sqrt{\lambda }} \,\, &\,\quad -\dfrac{\sin \varPsi (t,X_{3})}{\sqrt{\lambda }} \,\,&\,\quad - \dfrac{ \psi (t)}{H} r_{{\varvec{x}}} \sin \theta (t) \\ \dfrac{\sin \varPsi (t,X_{3})}{\sqrt{\lambda }} \, \, &\,\quad \,\dfrac{\cos \varPsi (t,X_{3})}{\sqrt{\lambda }}\, \, &\,\quad \, \dfrac{ \psi (t)}{H} r_{{\varvec{x}}} \cos \theta (t) \\ 0 &\,\quad 0 &\,\quad \lambda \end{array} \right), \end{aligned}$$

where angle $\theta$ is related to spatial position and is given by $\theta (t) = \arctan \left( {X_2}/{X_1}\right) + \varPsi (t,X_3)$ and $r_{{\varvec{x}}}$ is the radial position throughout the sample. On the top surface, the deformation metrics can be found by replacing $\varPsi (t,X_3)$ with $\psi (t)$.

In every test, torque measurements are acquired at the top plate level. Here, the torque on the top surface $\varGamma _t$ (as identified in Fig. 1) is computed as

12 $$\begin{aligned} {\varvec{\tau}} = \int _{\varGamma_{t}} {\varvec{r}} \times {\varvec{t}} {\text {d}}\varGamma = \int _{\varGamma_{t}} {\varvec{r}} \times ({\varvec{\sigma}} \cdot {\varvec{n}}) {\text{ d }} \varGamma _t, \end{aligned}$$

where ${\varvec{n}} = [0, 0, 1]^{\mathrm{T}}$ is the normal to the top surface. Since the rotational forces are acting in plane, symmetrically around the *Z*-axis, then the only nonzero torque component is

13 $$\begin{aligned} \tau _3 = \int _{\varGamma _t} r_1 \sigma _{23} - r_2 \sigma _{13} {\text { d}} \varGamma _t , \end{aligned}$$

where the Cauchy stress components can be found from Eq. 8. Although the hydrostatic pressure *P* does not affect the torque computation, note that its value can be retrieved due to the zero normal traction on the wall boundary $\varGamma _{\mathrm{w}}$. By combining Eqs. 5 and 8 into

$$\begin{aligned} {\varvec{\sigma }} = \varvec{F}( {\varvec{S}}_{\mathrm{e}} + D_t^\alpha {\varvec{S}}_{\mathrm{v}} )\varvec{F}^{\mathrm{T}} + JP{\varvec{I}}, \end{aligned}$$

and knowing that ${\varvec{t}} = {\varvec{\sigma }} \cdot {\varvec{n}} = {\varvec{0}}$, *P* can be determined by balancing out the elastic and viscoelastic components in the traction normal on the wall, $\varGamma _{\mathrm{w}}$.

### Constitutive modelling of liver tissue

In this study, the nonlinear liver behaviour is investigated under combined large compressions and shear strains. An example of angular displacements employed is shown in Fig. 1. For modelling purposes, it is assumed that in the reference configuration the bovine liver samples are stress free and isotropic. The observed torque behaviour is modelled using a viscoelastic adaption of three hyperelastic models commonly applied in soft tissue mechanics, with the aim of drawing a comparison between their suitability to model the data: polynomial (a modified form of the Mooney–Rivlin model, which will be indicated by *vMR*$^*$), Ogden (*vOG*) and exponential (*vEXP*).

#### Viscoelastic modified Mooney–Rivlin model

The simplest model considered here is a modified Mooney–Rivlin strain energy function, which comprises two parts: $W_{1}= (I_{\hat{ {\varvec{C}}}}-3)/2$ and $W_{2} = (II_{\hat{{\varvec{C}}}}-3)^2/8$. The first part is a linear neo-Hookean term, whereas the second term, compared to the original Mooney–Rivlin model, is quadratic, in order to trigger a more accentuated nonlinear response. This modified form has been previously employed in capturing polymer hyperelasticity (Capilnasiu et al. 2019), as the classical Mooney–Rivlin form was found to be unsuitable to model liver tissue hyperelasticity at large strains (Chui et al. 2004). In this form, the PK2 tensors are derived to be

14a $$\begin{aligned}&{\varvec{S}}_{\mathrm{e}}^1&= \dfrac{1}{J^{2/3}} \left( {\varvec{I}} - \dfrac{I_{\varvec{C}}}{3}\varvec{C}^{-1} \right) , \end{aligned}$$

14b $$\begin{aligned}&{\varvec{S}}_{\mathrm{e}}^2&= \dfrac{1}{J^{4/3}} \big ( II_{\hat{\varvec{C}}} - 3 \big ) \bigg ( \varvec{C}- \dfrac{II_{\varvec{C}}}{3}\varvec{C}^{-1} \bigg ). \end{aligned}$$

The above form provides a purely elastic part, with ${\varvec{S}}_{\mathrm{e}}^1$ leading to a linear response in shear and ${\varvec{S}}_{\mathrm{e}}^2$ leading to a nonlinear response. Viscoelasticity is introduced by taking a fractional-order derivative on the ${\varvec{S}}_{\mathrm{e}}^1$ tensor, as described in Eq. 5. Initially, a more extensive Mooney–Rivlin-based model was considered (“Appendix 1”, Eq. 31), but it did not perform much better than a model with fewer parameters. Hence, the total PK2 tensor for the *vMR*$^*$ law considered here is

15 $$\begin{aligned} {\varvec{S}} = C {\varvec{S}}_{\mathrm{e}}^2+ \delta D_t^ \alpha ({\varvec{S}}_{\mathrm{e}}^1) + {\varvec{S}}_{\mathrm{p}}, \end{aligned}$$

with *C*, $\delta$ (Pa), and $\alpha$ (unitless) being material parameters. Note that *C* and $\delta$ act as linear scalings on the model components, whereas $\alpha$ triggers a nonlinear response (hence it will be referred to as a nonlinear parameter, with the understanding that it leads to a nonlinear torque response).

#### Viscoelastic Ogden-based model

The second type of model considered is the Ogden model, described by the strain energy function $W= (\lambda _1^{b}+ \lambda _2^{b}+ \lambda _3^{b}-3)/(2b),$ and the corresponding PK2 tensor

16 $$\begin{aligned} {\varvec{S}}_{\mathrm{e}}^b = \sum \limits _{i=1}^ {3} \lambda _i^{b-1} {\varvec{v}}_i \otimes {\varvec{v}}_i. \end{aligned}$$

Here, power *b* is a nonlinear parameter (i.e. the torque depends nonlinearly on it), $\lambda _i$ are the three principal stretches, and ${\varvec{v}}_i$ are the corresponding eigenvectors of tensor ${\varvec{C}}$. A more comprehensive Ogden-based model was initially considered (“Appendix 1”, Eq. 32); however, it did not perform significantly better than a single-component model and made the parametrisation non-unique. Thus, the total PK2 stress considered here includes only a viscoelastic and a hydrostatic part:

17 $$\begin{aligned} {\varvec{S}} = \delta D_t^ \alpha ( {\varvec{S}}_{\mathrm{e}}^{b} )+ {\varvec{S}}_{\mathrm{p}}, \end{aligned}$$

where $\delta$ (Pa) is a linear scaling parameter, while the fractional-order derivative $\alpha$ and the eigenvalue power *b* (unitless) parameters act nonlinearly (and hence will be referred to as nonlinear parameters). This model will be referred to as the viscoelastic Ogden model *vOG*.

#### Viscoelastic exponential model

The last type of model considered here is the isotropic exponential Fung-type model, described by the strain energy function $W= (\exp (b(II_{{\varvec{C}}}-3))-1)/(4b)$. The corresponding elastic PK2 tensor is derived to be

18 $$\begin{aligned} {\varvec{S}}_{\mathrm{e}}^b = \exp (b (II_{{\varvec{C}}}-3)) {\varvec{C}} , \end{aligned}$$

with power *b* being a nonlinear scaling parameter. As before, viscoelasticity is introduced by the fractional-order derivative $D_t^ \alpha {\varvec{S}}_{\mathrm{e}}$. For this model, in order to ensure that the deviatoric and hydrostatic parts are separated after applying the time derivative, let the PK2 tensor be defined as

19 $$\begin{aligned} {\varvec{S}} = \delta {\text {Dev}} [D_t^\alpha {\varvec{S}}_{\mathrm{e}}^{b}] + {\varvec{S}}_{\mathrm{p}}, \end{aligned}$$

where $\delta$ (Pa) is a linear scaling parameters and *b* (unitless) is a parameter that acts nonlinearly. Hence, Eq. 19 defines the viscoelastic exponential model *vEXP*. A more comprehensive form of the exponential model was also considered (“Appendix 1”, Eq. 33). However, similarly to the Ogden-based model, the extensive form did not perform significantly better and it led to non-unique parameter identification.

### Data analysis and model fitting

Parameters from all models were tuned to match the experimental data. In this case, torque measurements ($\tau ^d$) were compared against the torque corresponding to the models ($\tau ^m$). In order to obtain $\tau ^m$, the PK2 stress tensor for each individual model (*vMR*$^*$—Eq. 15, *vOG*—Eqs. 17, and *vEXP*—Eq. 19) was used in Eq. 8 in order to quantify the Cauchy stress and in Eq. 13 to quantify the torque. Then, the model parameters were adjusted to match the data by solving a minimisation problem. Three different objective functions were employed, which will be described later in this section.

#### Minimisation problem

Let ${\varvec{y}}$ denote a set of *M* parameters that matches a model to the data. In order to find ${\varvec{y}}$, a minimisation problem of the form

20 $$\begin{aligned} \theta = {\mathop {{{\,\mathrm{arg\,min}\,}}}\limits _{{\varvec{y}} \in {\mathbb {R}}^M_{+}}} {\mathcal {J}}({\varvec{y}}, {\varvec{\tau }}^d) \end{aligned}$$

is posed, where ${\mathcal {J}}$ is a function to be minimised. For the models presented here, ${\varvec{y}}$ comprises the nonlinear parameter $\alpha$ and, where applicable, *b*, *C* and $\delta$. However, the *m* linear parameters can simply be found by inverting a system of linear equations, as it will be seen shortly. This leads to a simplified minimisation problem

21 $$\begin{aligned} \theta ^* = {\mathop {{{\,\mathrm{arg\,min}\,}}}\limits _{{{\varvec{y}}}^* \in {\mathbb {R}}^{M-m}_+}} {\mathcal {J}^*}({\varvec{y}}^*, {\varvec{\tau }}^d), \end{aligned}$$

where ${\varvec{y}}^*$ spans the nonlinear parameters only ($\alpha$ and, if applicable, *b*).

In this work, the minimisation problem $\theta ^*$ is solved by carrying out a parameter sweep over the nonlinear parameters. Specifically, the fractional order $\alpha$ is iterated between 0.05 and 1 (with a step of 0.05), to ensure that the whole spectrum from elastic to viscous is captured. In the interval [0.15, 0.4], which is close to the literature range estimated for $\alpha$, a finer step of 0.01 was used. Similarly, power *b* is examined over a range—[0.1, 14] for the *vOG* model and [0.1, 3] for the *vEXP* model, with coarse refinements, to see trends. Then, we focused on the range [1, 14] for the *vOG* model, with refinements of 0.5, and on [1, 1.5] for the *vEXP* model, with refinements of 0.1, to isolate parameter values.

For each combination of $\alpha$ and *b* considered, the best linear parameters *C* and $\delta$ can be found by solving a linear system of equations

22 $$\begin{aligned} {\varvec{A}} {\varvec{x}} = {\varvec{b}}, \end{aligned}$$

with the understanding that ${\varvec{A}} = {\varvec{A}}(\alpha , b)$ and ${\varvec{x}} = {\varvec{x}}(\alpha , b)$. The matrix ${\varvec{A}}$ and vector ${\varvec{b}}$

23 $$\begin{aligned} {\varvec{A}} = \left[ \begin{array}{c c} \left[ \tau ^{m,e}_1 \right. &\, \left. \tau ^{m,v}_1 \right] \\ \left[ \tau ^{m,e}_2 \right. &\, \left. \tau ^{m,v}_2 \right] \\ \vdots &\, \vdots \\ \left[ \tau ^{m,e}_N \right. &\, \left. \tau ^{m,v}_N \right] \\ \end{array} \right] \qquad {\varvec{b}} = \left[ \begin{array}{c} \left[ \tau ^{d}_1 \right] \\ \left[ \tau ^{d}_2 \right] \\ \vdots \\ \left[ \tau ^{d}_N \right] \\ \end{array} \right] \end{aligned}$$

comprise the elastic (if applicable) and viscoelastic torque model components and the torque data measurements, respectively. Subscripts 1 to *N* indicate the tests considered, which are vertically concatenated. Each block $\left[ \tau ^{m,e}_i \, \tau ^{m,v}_i \right]$ comprises multiple time points. Vector ${\varvec{x}} = [C \, \, \delta ]^{\mathrm{T}}$ contains the unknown linear parameters to be found. For further use throughout the section, subscript *i* denotes the block corresponding to test *i*, i.e.

$$\begin{aligned} {\varvec{A}}_i = \begin{array}{cc} \left[ \tau ^{m,e}_i \right.&\left. \tau ^{m,v}_i \right] \end{array} \hbox { and }{\varvec{b}}_i = \left[ \tau ^{d}_i \right] . \end{aligned}$$

The model components $\tau ^{m}$ were computed across a circular surface of radius *r*, at time points corresponding to the data readings. Spatial integration of the modelled torque across the top face of the cylindrical sample was carried out using a triangular mesh with 765 elements. For the time integration, a discrete time step was set so that it matches the data points, using ${\mathrm{d}}t = \frac{1}{fT}$, where *f* is the frequency and *T* is the number of points in an oscillatory period. Specifically, ${\mathrm{d}}t =$ 0.0059, 0.0049 and 0.0049 s for the samples at 0.5, 1 and 2 Hz, respectively.

Three different norms were employed in order to quantify the model fit to the data. Firstly, the classical $L_2$ norm is investigated. Further on, a point-wise scaling norm is introduced, which is an adaption of the $L_2$ norm. Lastly, a parameter scaling norm is designed, where some constraints on the linear parameters are relaxed. The norms presented are constructed such that the error is 0% for a perfect fit and 100% when the linear parameters are set to 0 Pa. The contrasting nature of the norms leads to gathering different insights about the data and thus contributes to an overall better understanding of the liver tissue.

#### $L_2$ norm

The first norm relies on the MATLAB-implemented linear solver “lsqnonneg”. The linear parameters are found by minimising the remainder in a least squares (lsq) sense, using the $L_2$ norm ($||\cdot ||_2$), i.e.

24 $$\begin{aligned} {\mathcal {J}}^*_{\mathrm{lsq}}=\dfrac{ \left| \left| {\varvec{A}} {\varvec{x}} - {\varvec{b}} \right| \right| _2}{||{\varvec{b}} ||_2 }. \end{aligned}$$

While straightforward, this norm favours the tests that employ larger deformations and thus attain higher torque amplitudes.

#### Point-wise norm

An alternative to the classic $L_2$ norm is to scale values to ensure that all points carry equal importance in the fitting process. To achieve this, each point in the data and model is scaled by its corresponding amplitude in the data (point-wise scaling). In general form, this can be written as

25 $$\begin{aligned} ({\varvec{a}}; {\varvec{b}})_{pw} = \sum \limits _k \left( \dfrac{a_k}{\max ({\mathrm{tol}}, |b_k|)} \right) ^{2}, \end{aligned}$$

where tol is a nonzero tolerance level, to avoid division by zero. Thus, the error measure, which is also the function to be minimised, becomes

26 $$\begin{aligned} {\mathcal {J}}\,^{*}_{pw} = \dfrac{\left( \sum \limits _i \left( {\varvec{A}}_i {\varvec{x}} - {\varvec{b}}_i; {\varvec{b}}_i \right) _{pw} \right) ^{1/2}}{\left( \sum \limits _i ({\varvec{b}}_i; {\varvec{b}}_i)_{pw} \right) ^{1/2}}. \end{aligned}$$

This adapted norm leads to potentially higher $L_2$ norm errors, but it also ensures that the curve trends (e.g. the non-sinusoidal torque response in Fig. 1) are better matched, irrespective of their amplitude.

#### Parameter scaling norm

Parameter variability can be encountered when analysing samples collected from different livers or different locations in the liver. Furthermore, the parametrisation process can be affected by shear softening—an effect which might be observed when a material is sheared at successive increasing levels (Perepelyuk et al. 2016). Here, in order to accommodate for parameter variability due to sample location or shear softening, we introduce a norm that allows for flexibility in the linear scaling parameters.

Since a single frequency was investigated per sample (either 0.5, 1 or 2 Hz), this is not sufficient to allow for variability in the fractional order $\alpha$. Moreover, it is observed that the shape of the data curves tends to be preserved across samples, whereas the amplitude scaling changes. Hence, presuming that the nonlinear parameters govern the shape of the torque curves, $\alpha$ and *b* (if applicable) are assumed to be consistent across samples and shear softening. By contrast, the linear scaling parameters *C* and $\delta$ are assumed to vary, and their distribution will be examined in order to understand the amplitude behaviour of the data.

Firstly, consider the normalised

$$\begin{aligned} {\varvec{A}}_i^* = \begin{array}{cc} \left[ \dfrac{\tau ^{m,e}_i}{n_i \left| \left| \tau ^d_i \right| \right| _2 } \right.&\left. \dfrac{\tau ^{m,v}_i}{n_i \left| \left| \tau ^d_i \right| \right| _2 } \right] \end{array} {\text { and }}{\varvec{b}}_i^* = \left[ \dfrac{\tau ^{d}_i}{n_i \left| \left| \tau ^d_i \right| \right| _2 } \right] \end{aligned}$$

which make up normalised matrix ${\varvec{A}}^*$ and vector ${\varvec{b}}^*$. Here, $n_i$ is the number of data points in test *i*. Additionally, let

27 $$\begin{aligned} {\varvec{x}}^* = {\varvec{x}}_i \beta _i \end{aligned}$$

be a set of unique linear parameters $[C^* \, \delta ^*]$ which can be transformed into the test-specific parameters ${\varvec{x}}_i$ by employing the scaling $\beta _i$. In order to find ${\varvec{x}}^*$ and $\beta$, an iterative process is employed, starting with each $\beta _i = 1$:

28 $$\begin{aligned} {\varvec{x}}^*= & \, {\mathop {{{\,\mathrm{arg\,min}\,}}}\limits _{{\varvec{x}}^*}} \left( \sum \limits _{i=1}^{N} \left| \left| {\varvec{A}}^*_i {\varvec{x}}^* / \beta _i - {\varvec{b}}^*_i \right| \right| _2^2 \right) ^{1/2} , \end{aligned}$$

29 $$\begin{aligned} \beta _i= & \, {\mathop {{{\,\mathrm{arg\,min}\,}}}\limits _{\beta _i}} \left| \left| {\varvec{A}}^*_i {\varvec{x}}^* - {\varvec{b}}^*_i \beta _i \right| \right| _2 . \end{aligned}$$

The above sequence of minimisation equations is repeated until there is no change in the error ${\mathcal {J}}_{sc}^*$,

30 $$\begin{aligned} {\mathcal {J}}\,^{*}_{sc} = \dfrac{ \left( \sum \nolimits _{i=1}^{N} \left| \left| {\varvec{A}}^*_i {\varvec{x}}^* / \beta _i - {\varvec{b}}^*_i \right| \right| _2^2 \right) ^{1/2} }{ \left| \left| {\varvec{b}}^* \right| \right| _2} . \end{aligned}$$

Although the set of linear parameters ${\varvec{x}}_i$ is different for every test considered, note that this is different than solving each test individually, as this norm ensures that the nonlinear parameters are fixed across all tests. Solving each test individually would yield the similar results only if $\alpha$ and *b* were known a priori.

## Results

This work is based on data acquired at a range of compression preloads, oscillatory shear strains and frequencies. At compression preloads of 1%, 10% and 20%, the averaged measured normal force is 0.07 N, 0.2 N and 0.7 N. At the smallest shear strain (1%), the data are close to linear viscoelasticity, with the nonlinearity becoming more evident at increased shear strains. Increasing the frequency also enhances the nonlinear behaviour of the liver tissue samples. Hysteresis effects manifest during all tests employed, and they tend to increase slightly with increasing shear strain. Strain rate dependence is observed, as both nonlinearity and hysteresis increase with frequency. These observations point towards a strain rate dependent, nonlinearly viscoelastic liver tissue behaviour.

**Fig. 2:**
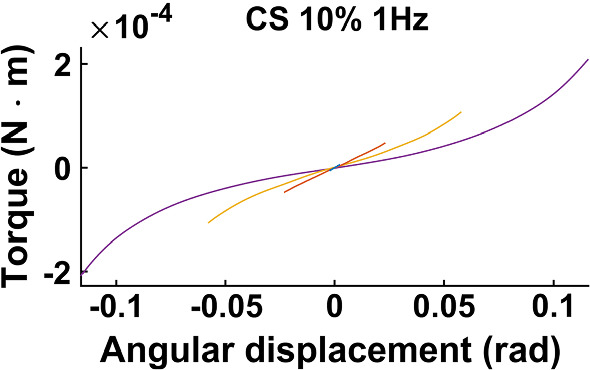
Mean loading and unloading torque response versus angular displacement at 1 Hz, CS 10%

When investigating the acquired data, a strain softening effect is observed. That is, the more strained the tissue sample is, the less force is required to produce a strain increment. This is exemplified in Fig. 2 for shear strain (CS 10%, 1Hz), but a similar trend is exhibited for compressive strain as well. The curves capture the mean loading and unloading response, and it can be seen that the tangent of the torque versus shear strain decreases as the maximum strain amplitude increases.

### Viscoelastic models tailored with the $L_2$ norm

**Fig. 3:**
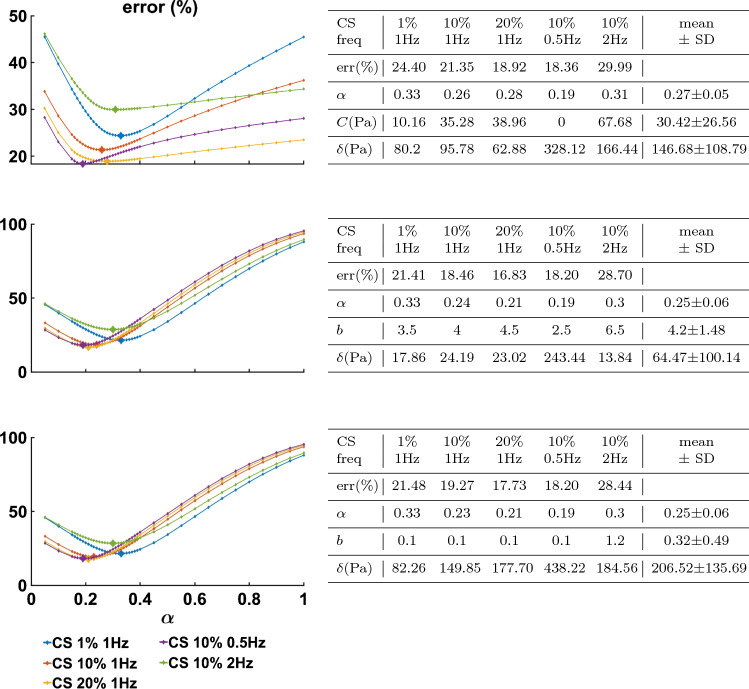
The minimum error for the $L_2$ norm (Eq. 24), obtained for each data group, for fractional order $\alpha$ values between 0.05 and 1. Plots show minimal error obtained for *vMR*$^*$ (Top), *vOG* (Middle) and *vEXP* (Bottom) models, with the larger-sized markers being obtained for the parameters presented in the corresponding tables

Figure 3 presents the $L_2$ error norm for each $\alpha$ value investigated for the optimal set of all remaining parameters, with larger markers denoting the minimum. The data were grouped by compression strain and frequency, with each group comprising the corresponding shear strain tests (e.g. CS 10%, 1 Hz, $\gamma = 1, 10, 25, 50\%$). The best parameter set for each data group is presented in the corresponding tables.

**Fig. 4:**
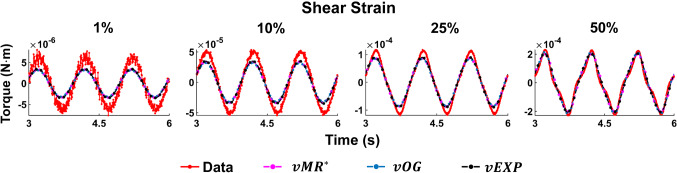
Example of the three models fit to the data acquired at 1 Hz, compression strain 10%, using the $L_2$ norm. The parameters employed to produce the models’ curves can be found in column 2 (CS 10% 1 Hz) of the corresponding tables in Fig. 3

**Fig. 5:**
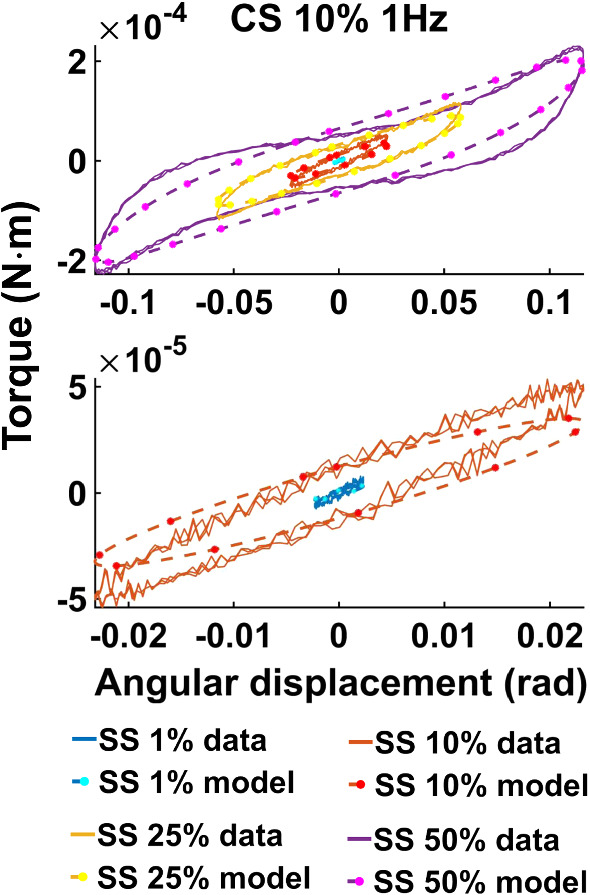
Lissajous curve exemplifying the *vEXP* model fit to the data acquired at 1 Hz, compression strain 10%. The parameters employed to produce the model’s curves can be found in column 2 (CS 10% 1 Hz) of the bottom table in Fig. 3. The curves corresponding to shear strains 1–10–25–50% are shown in the top quadrant, while the lower quadrant zooms on shear strains 1–10%

Figure 4 illustrates examples of the three models—*vMR*$^*$, *vOG* and *vEXP*—fit to the data tests at 1 Hz and CS 10%. The three model curves were produced using the best fit parameters obtained when fitting the whole shear strain range (1–50%). Figure 5 shows the Lissajous plots (torque readings depending on the angular displacement) corresponding to CS 10%, 1Hz. The continuous lines show the data readings, while the dashed lines show the *vEXP* model fit.

### Viscoelastic models tailored with the point-wise norm

**Fig. 6:**
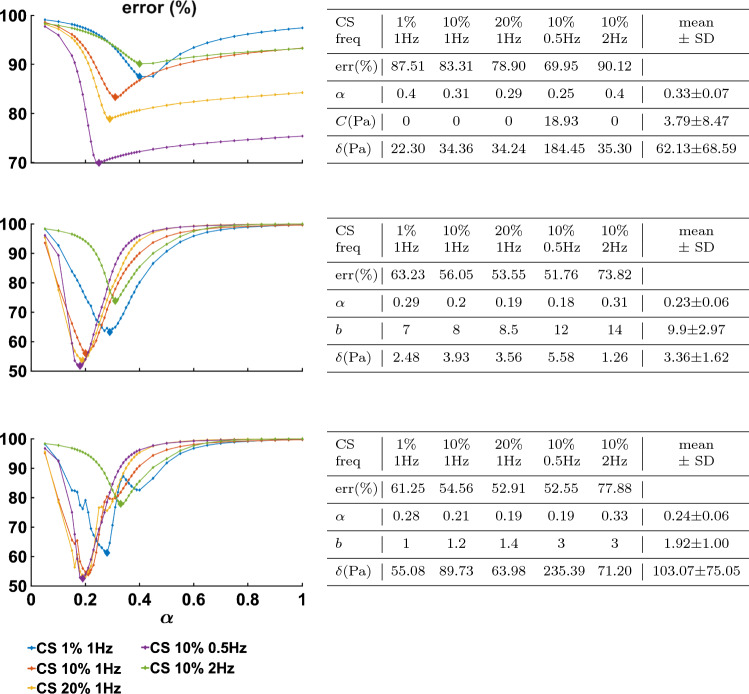
Minimum error for the point-wise scaling norm (Eq. 26), obtained for each data group, for fractional-order $\alpha$ values between 0.05 and 1. Plots show minimal error obtained for *vMR*$^*$ (Top), *vOG* (Middle) and *vEXP* (Bottom) models, with the larger-sized markers being obtained for the parameters presented in the corresponding tables

The point-wise error norm behaviour with $\alpha$ is shown in Fig. 6 for the *vMR*$^*$ (top), *vOG* (middle) and *vEXP* (bottom) models. The larger error markers identify the overall best parameter fit, obtained for the parameters presented in the corresponding tables.

**Fig. 7:**
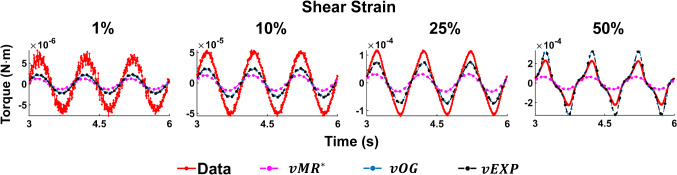
Example of the three models fit to the data acquired at 1 Hz, compression strain 10%, using the point-wise scaling norm. The parameters employed to produce the models’ curves can be found in column 2 (CS 10% 1 Hz) of the corresponding tables in Fig. 6

**Fig. 8:**
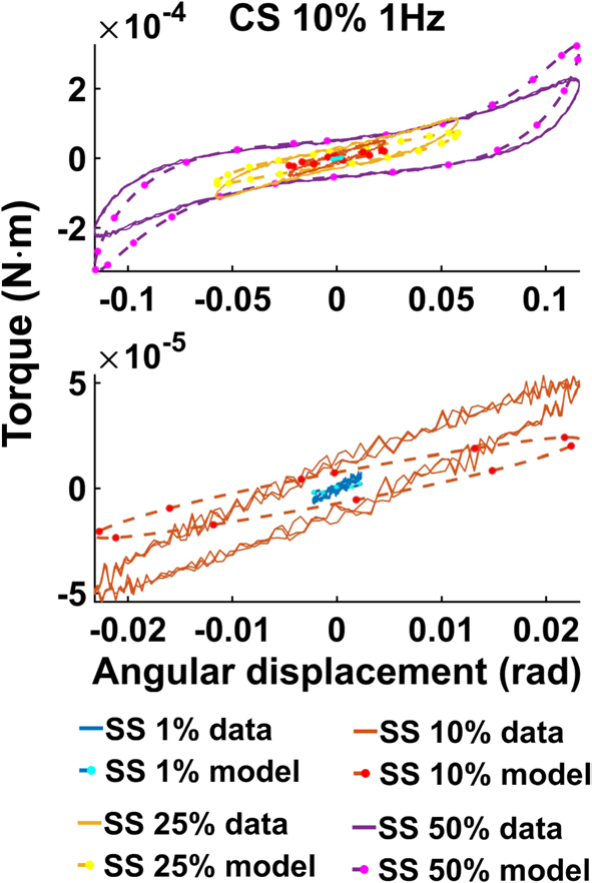
Lissajous curve exemplifying the *vEXP* model fit to the data acquired at 1 Hz, compression strain 10%. The parameters employed to produce the model’s curves can be found in column 2 (CS 10% 1 Hz) of the bottom table in Fig. 6. The curves corresponding to shear strains 1–10–25–50% are shown in the top quadrant, while the lower quadrant zooms on shear strains 1–10%

Figure 7 presents the best models fit to the data acquired at 1 Hz, CS 10%. The models’ parameters can be found in Fig. 6. The same dataset and the *vEXP* model fit are also conveyed in Lissajous curves in Fig. 8.

### Viscoelastic models tailored with the parameter scaling norm

**Fig. 9:**
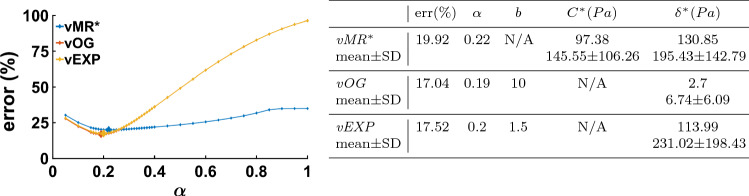
Minimum model error (*vMR*$^*$, *vOG* and *vEXP*) for the parameter scaling norm (Eq. 30), obtained for the tests altogether, for fractional-order $\alpha$ values between 0.05 and 1. The minimal error across $\alpha$ is enhanced by the larger-sized marker, obtained for the parameters presented in the corresponding table

Figure 9 shows the minimum parameter scaling error norm corresponding to the models (*vMR*$^*$, *vOG*, *vEXP*), for every fractional order $\alpha$ investigated. All 18 tests were considered simultaneously, hence a single curve corresponding to each model, compared to the previous analogue Figs. 3 and 6, where subgroups of the data tests were considered. The smallest error across the $\alpha$ range is identified by the larger markers, and the set of parameters corresponding to these minima are found in the corresponding table.

**Fig. 10:**
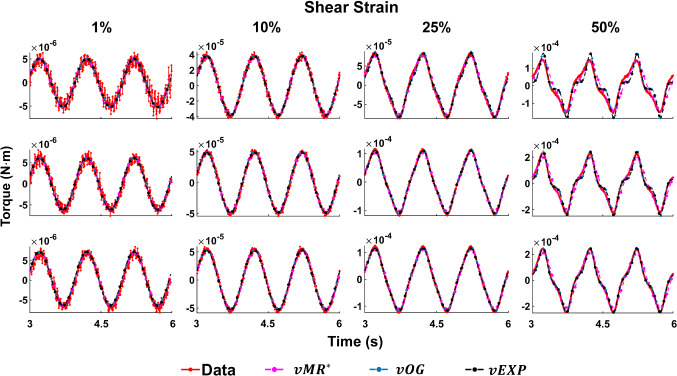
Three models fit to the data acquired at 1 Hz, using the parameter scaling norm. The first row illustrates the tests at CS 1%, the second row at CS 10% and the third row at CS 20%. The parameters employed to produce the models’ curves can be found in the table corresponding to Fig. 9

**Fig. 11:**
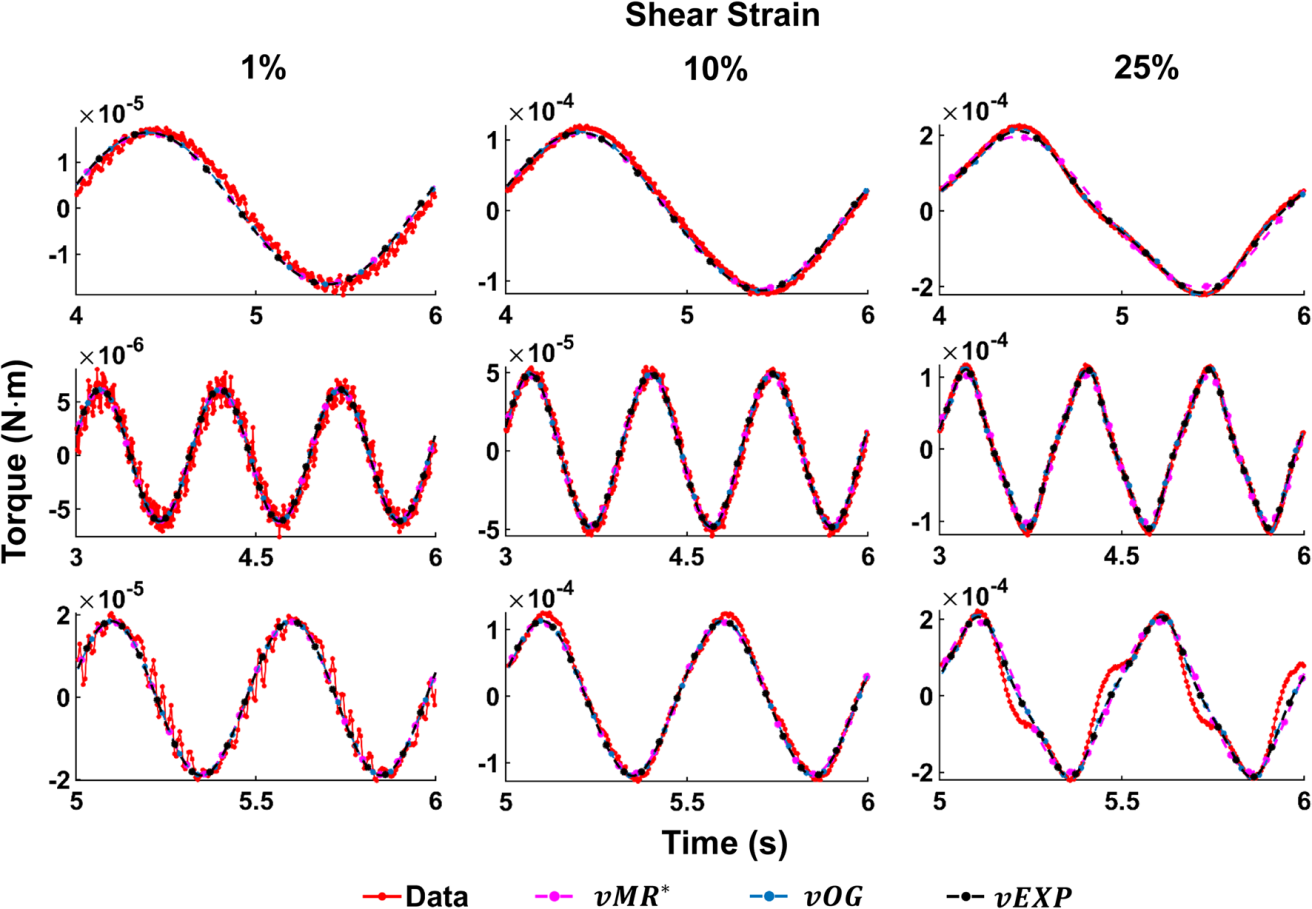
Three models fit to the data acquired at CS 10%, using the parameter scaling norm. The first row illustrates the tests at 0.5 Hz, the second row at 1 Hz and the third row at 2 Hz. The parameters employed to produce the models’ curves can be found in the table corresponding to Fig. 9

**Fig. 12:**
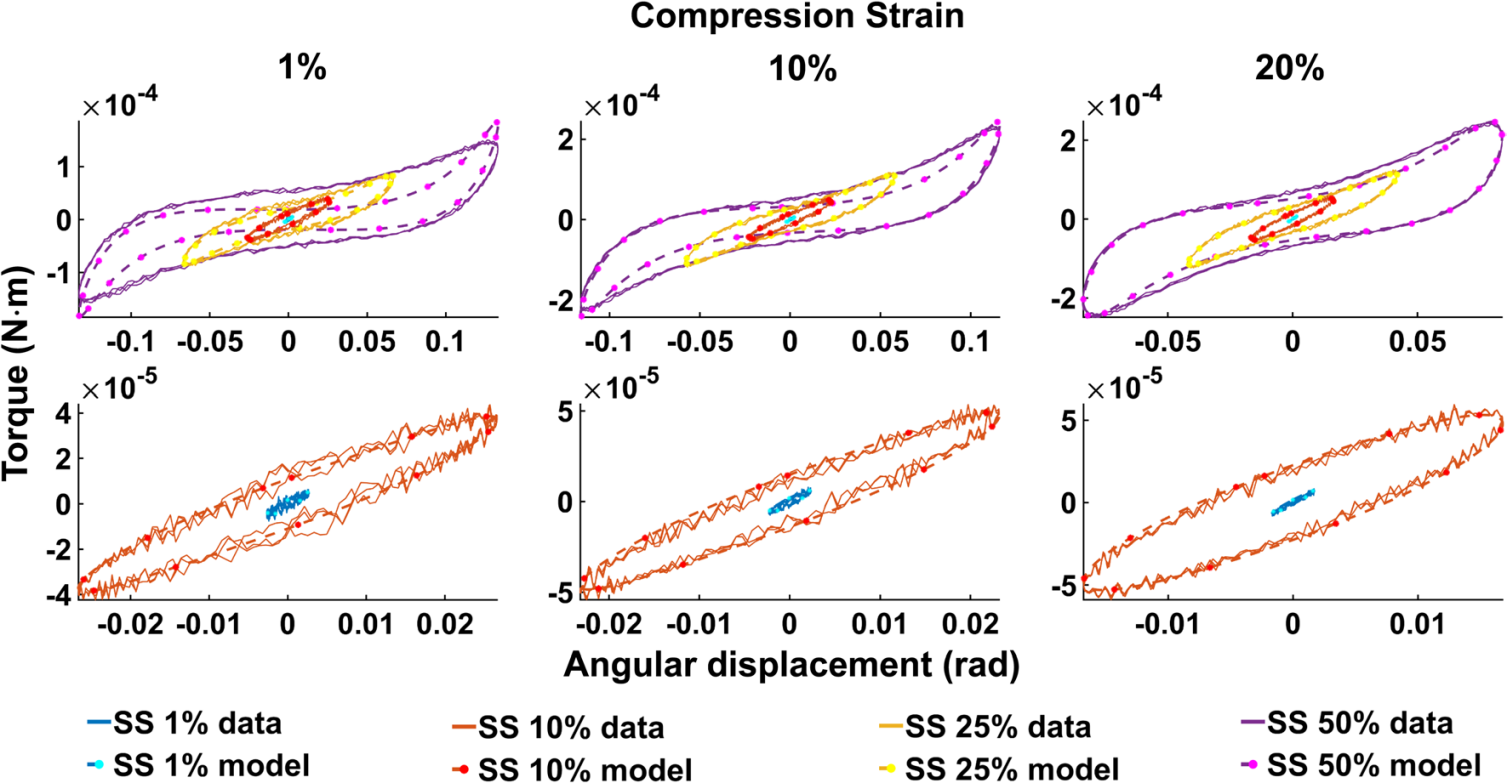
Lissajous curve exemplifying the *vEXP* model fit to the data acquired at 1 Hz. The parameters employed to produce the model’s curves can be found in the table corresponding to Fig. 9. The top row shows the curves corresponding to shear strains 1–10–25–50%, while the bottom row zooms on shear strains 1–10%. Compression strain increases from left (1%) to middle (10%) and right (20%)

**Fig. 13:**
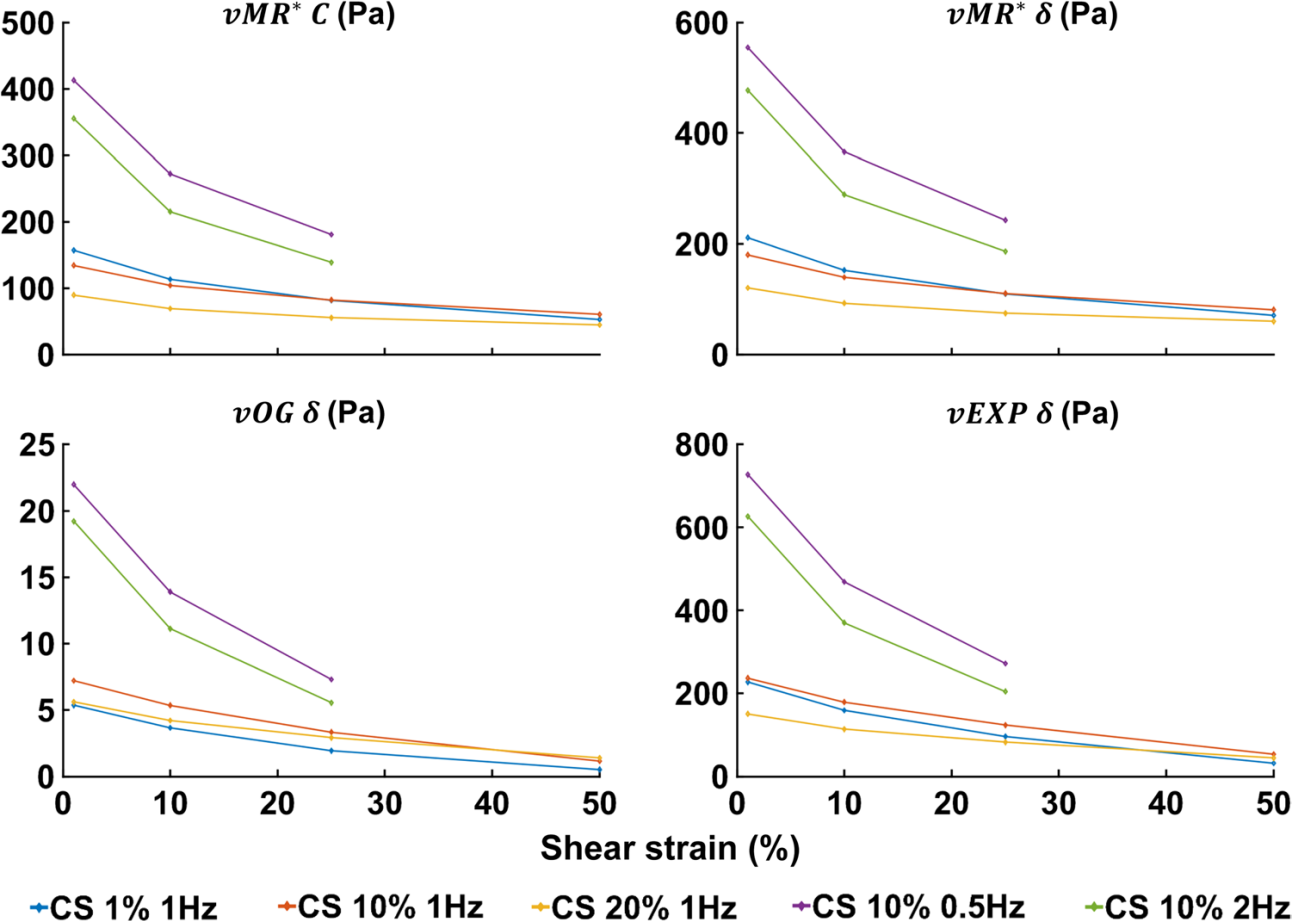
Absolute linear parameters for the models, obtained by transforming the starred$^*$ relative parameters into their absolute counterparts using Eq. 27. The top panels show the *C* and $\delta$ parameters scaling the elastic and viscoelastic components of the *vMR*$^*$ model. The bottom panels show the $\delta$ parameter scaling the viscoelastic component of the *vOG* and *vEXP* models, respectively

Figures 10 and 11 show all data tests acquired, and the three model fits. Figure 10 shows all datasets acquired at a frequency of 1 Hz, while Fig. 11 shows datasets acquired under a compression strain of 10%. Example Lissajous curves are shown, for the data and *vEXP* model at 1 Hz, in Fig. 12. The top row shows all four shear strains (1–10–25–50%), while the bottom row zooms in on the 1 and 10% shear strains. Compression strain increases from the left column (1%) to middle (10%) and right (20%). In these figures, the models’ curves were obtained by employing the starred parameters from Fig. 9, scaled according to Eq. 27. The test-specific linear parameters can be seen, for all models, in Fig. 13, and their mean and standard deviation are shown in Fig. 9.

### Viscoelastic models tailored with the parameter scaling norm in the case of non-ideal compression

**Fig. 14:**
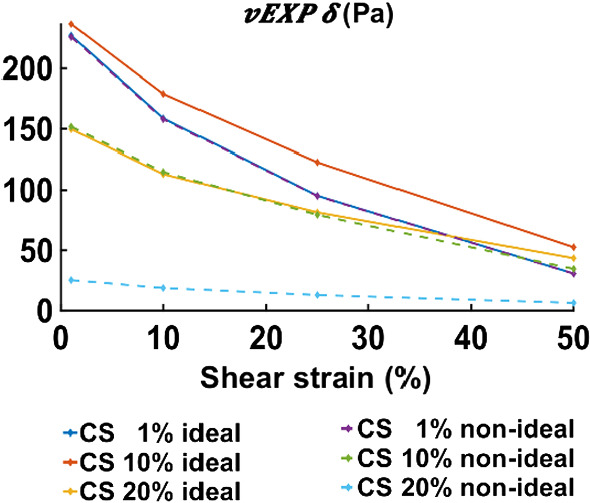
Absolute linear parameters for the *vEXP* model, corresponding to the data at 1 Hz. The solid lines (blue, orange, yellow) indicate the value of the $\delta$ parameter when using ideal deformation assumption and are the same as the ones presented in Fig. 13, bottom right panel; the dashed lines (purple, green, cyan) indicate the $\delta$ parameter when using non-ideal compression. The non-ideal parameters were obtained by scaling the ideal parameters by the values presented in Table 2

In this study, for rapid model evaluation, the analysis assumed ideal compression. However, the testing conditions (serrated plates) actually led to non-ideal compression and hence a barrelling of the tissue samples. “Appendix 2” presents details on the differences observed between ideal and non-ideal compression, and it reveals that, for torque, a scaling factor needs to be applied in order to correct the linear parameters. For the *vEXP* model, for the data at 1 Hz, the correcting factors are presented in Table 2, and the corrected parameters are shown in Fig. 14.

**Table 2:** Multiplying factors between ideal and non-ideal torque and normal force measurements

Torque	SS 1%	SS 10%	SS 25%	SS 50%
CS 1%	1.0050	1.0046	1.0031	1.0000
	0.2041%	0.2026%	0.2043%	0.1730%
CS 10%	1.5560	1.5568	1.5460	1.5123
	0.6844%	0.7326%	0.9007%	1.1374%
CS 20%	5.9433	6.0363	6.3176	6.8061
	3.0972%	2.7304%	1.9302%	1.5789%

## Discussion

When comparing the three models (*vMR*$^*$, *vOG* and *vEXP*) within the $L_2$ norm performance, it can be seen that the errors presented in Fig. 3 are very similar, with the *vMR*$^*$ model errors being slightly higher (at most $\approx 3\%$). This is likely due to the fact that the other two models are more suitable to capture the nonlinear trends. However, it can be seen from Fig. 4 that all three models match the closest data peaks at shear strain 50%. This is because the data in this study can vary even by two orders of magnitude, with the higher amplitude data having the largest impact on the $L_2$ norm. As a result, amplitudes in the other tests have lesser fidelity, as a consequence of the $L_2$ norm, which penalises less discrepancies at smaller amplitudes. This mismatch is further accentuated by the strain softening effect. It can be seen that the data exhibit a shallower increase in amplitude with shear strain (1–50%) than the models are. The bias of the $L_2$ norm towards the higher amplitude points can also be observed in Fig. 5, where the *vEXP* model fit well the peak at shear strain 50%, yet the rest of the model torque curve looks idealised (almost perfectly elliptic) and does not follow the data curve.

In order for the models to better follow the data curve trends throughout the entire deformation, a point-wise scaling norm (Eq. 26) was designed, which penalises discrepancies between data and models, across all points considered, evenly. For this norm, the errors for the *vOG* and *vEXP* models are similar, with the *vMR*$^*$ model performing significantly worse ($\approx 20\%$, as shown in Fig. 6). This is because the *vMR*$^*$ model cannot recreate the nonlinear trends exhibited. Hence, this norm drives the model peak points to match closely the lower amplitude data points (Fig. 7), by forcing a delayed response (higher $\alpha$ values, as shown in the top table of Fig. 6, compared to the middle and bottom tables).

Figure 7 shows that the *vOG* and *vEXP* models are able to capture the data trends; however, there is a data-model amplitude discrepancy for all tests. This discrepancy is more accentuated than for the $L_2$ norm (Fig. 4) because the models employ a higher nonlinear parameter *b*—8 to 4 for *vOG*, as shown in the middle tables of Figs. 3 and  6, and 1.2 to 0.1 for *vEXP*, as shown in the bottom tables of the same figures. This determines a steeper increase in the models’ amplitude. Figure 8 clearly shows that this norm leads to a better model match of the data curve trend, at the cost of discrepancy in the peaks.

In the models, the nonlinear parameter $\alpha$ determines the phase delay between the shear strain input and torque output. The nonlinear parameter *b* controls the shape and amplitude of the torque curve, where $b=0$ leads to a linear response (i.e. sinusoidal output as a result of a sinusoidal input) and higher values lead to an increased nonlinear response. The linear parameters *C* and $\delta$ amplify the contribution of the model components. Comparing the results of the $L_2$ and point-wise norms, it can be concluded that the data curve trends can only be captured by accentuating the nonlinear characteristics of the models (e.g. increasing the *b* parameter). However, this deteriorates the peak amplitude match, as the data exhibit strain softening (Fig. 2).

Due to the fact that the data could not be described using a set of fully consistent parameters, the parameter scaling norm was designed so that the set of linear parameters can be adjusted according to each test, by scaling the models’ amplitude response. Hence, it is expected that the errors, computed using Eq. 30, are small. Indeed, as per Fig. 9, the parameter scaling norm leads to the smallest quantified errors, compared to the $L_2$ and point-wise scaling norms. Comparing the three models, it can be observed that error behaviour with $\alpha$ (Fig. 9) is almost identical for the *vOG* and *vEXP* models. For the *vMR*$^*$ model, the minimum error curve is shallow for $\alpha$ values between 0.15 and 0.4, leading to a less precise parameter identifiability.

With the three models reaching a similar minimum error, it is expected that they behave similarly, as shown in Figs. 10 and 11. The *vOG* and *vEXP* models are better at capturing the data nonlinearity, which becomes apparent at shear strains above 10%, as the curves start deviating from a pure sinusoidal wave, looking like a combination of triangle and sine waves. Although it appears that an increased compression strain simplifies the appearance of the shear strain torque curve, in reality it further complicates the nonlinear behaviour exposed due to shear straining. This complex behaviour is generally better captured by the *vOG* and *vEXP* models, although one notable test where the *vMR*$^*$ model performs better is at compression strain 1%, shear strain 50%, 1 Hz (top right panel in Fig. 10). There, *vMR*$^*$ captures the data amplitude and does not exhibit the exaggerated nonlinearity of the other two models; however, it performs inferiorly in other tests (e.g. compression strain 10%, shear strain 25%, 0.5 Hz—top right panel in Fig. 11).

The models’ curves in Figs. 10, 11 and 12 were produced using the parameters presented in Fig. 9. All three models employ a similar fractional-order derivative, between 0.19 and 0.22, which is in good agreement with the literature. It is notable that for the *vOG* and *vEXP* models, the nonlinear parameters $\alpha$ and *b* presented in Fig. 9 are similar to the parameter means presented in Fig. 6, obtained using the point-wise norm. This reinforces the suitability of the parameter scaling norm to be employed with this study’s data, as its main effect is facilitating the amplitude match.

The linear parameters $\delta ^*$ and, if applicable, $C^*$, are scaled according to each data test, leading to the test-specific parameters depicted in Fig. 13. It can be observed that for all three models, the linear parameters decrease with increasing shear strain, which reflects the shear strain softening effect. Interestingly, it appears that three data groups can be identified, which correspond to the frequency groups—0.5, 1 and 2 Hz. Since the linear parameters do not have a monotonic trend with frequency, with the parameters at 0.5 and 2 Hz being larger than the ones at 1 Hz, it is unlikely that this reflects a parameter–frequency coupling. Instead, this might be explained by a dependency on the sample location, as different samples were tested across the frequency tests. Additionally, the standard deviation of these parameters reaches up to 90% of their mean, as presented in Fig. 9, indicating a high linear parameter variability introduced by sample location and strain softening.

The effect of barrelling, due to non-ideal compression, was investigated in “Appendix 2”. For the *vEXP* model simulating the data at 1Hz, the corrected parameters can be seen in Fig. 14. The parameters at CS 1% remain similar between ideal and non-ideal compression, which is expected given the small compressive strain level. The parameters at CS 10% are downscaled by a factor of $\approx$ 1.5, according to Table 2, and maintain a similar softening trend. The parameters at CS 20% are downscaled according to the factors presented in Table 2. Although the non-ideal parameters trend appears flatter, this is because of the scale used (0 to 230), and in reality the softening trend is accentuated.

### Liver biomechanical considerations in the context of existing literature

In this study, polynomial, Ogden and exponential model forms were used. These types of models are commonly employed for capturing the nonlinear hyperelastic behaviour exhibited by tissue at large strains (Veronda and Westmann 1970; Zobitz et al. 2001; Chui et al. 2004; Gao et al. 2010). Polynomial models introduce nonlinearity through quadratic or higher-order terms, yet Ogden and exponential models are generally better suited in capturing the nonlinearity. However, loading–unloading tests reveal that biological tissues are viscoelastic (Jordan et al. 2009) and strain rate dependent (Liu and Bilston 2000; Miller 2000). Here, fractional derivative adaptions of the Mooney–Rivlin, Ogden and exponential models were introduced in order to capture the viscoelastic, strain rate-dependent behaviour of liver tissue. The fractional order $\alpha$ can vary from 0 (purely elastic response) to 1 (purely viscous response), with intermediate values leading to viscoelasticity. For fractional models, a frequency dependence of the type $\omega ^\alpha$ Holm and Sinkus (2010) is achieved. Considering the above, the *vMR*$^*$, *vOG* and *vEXP* models are appropriate for modelling the torque data in this study, which exhibits a frequency-dependent, nonlinear viscoelastic behaviour.

In a study similar to the present one, conducted by Ayyildiz et al. (2015), cylindrical liver samples of similar dimensions were investigated at a range of compression strains, shear strains and frequencies. Specifically, the authors investigated the effect of strain rate on compressions up to 20%, the influence of compressive preload (5–20%) on shear strains of 0.1–5% at a fixed frequency of 10Hz, and the combined effect of compressive preload (5–20%) and frequency sweep (0.1–10 Hz) on the shear modulus measured using 0.5% shear strain. It was found that the compressive preload affects the measured shear modulus, which is consistent with our findings [and the original findings in Tan et al. (2013)], since the compression strain influences the torque response measured, as shown in Fig. 10. Similarly, the frequency increase accentuates the nonlinear shear response (Fig. 11), which would correspond to an increased shear modulus, as indicated by Ayyildiz. As the detailed results on the shear strain sweep (0.1–5%) are not presented, the studies cannot be compared in this regard. To model the data, Ayyildiz used a generalised Maxwell model with 13 parameters, which captures the strain-dependent, nonlinear viscoelastic behaviour of the liver. Here, we used fractional viscoelasticity, which helps reducing the number of rheological elements considered and hence the number of model parameters.

### Study overview

In this work, three constitutive equations were proposed for modelling the behaviour of liver tissue under a range of deformations and frequencies, being parametrised by three error norms. For the data employed here, the $L_2$ norm proved to be unsuitable, as it was biased by the larger amplitude points. However, this is a straightforward norm that is convenient to use when analysing data of comparable magnitude. The point-wise norm was introduced in order to equalise the weight of each data point in the models’ fitting process. The *vMR*$^*$ model performed particularly poor with this norm; however, the *vOG* and *vEXP* models followed the data curve trends better than for the $L_2$ norm. Hence, this norm offered better insights into the nonlinearity of the data. With the shear strain softening leading to amplitude mismatches, the parameter scaling norm was designed to allow for flexibility in the set of linear parameters and thus facilitate the model to data amplitude match. In this case, the models’ fit was significantly improved, with all three models performing similarly in capturing the data particularities.

Based on the results of this study, the choice of constitutive law for modelling the liver behaviour depends on the complexity desired. The *vMR*$^*$ model provided a marginally less suitable fit compared to the *vOG* and *vEXP* models; however, it is very practical due to its simple form, requiring only the computation of invariants and identifying a single nonlinear parameter. Nevertheless, for the data presented here, the shallow minima of the error norm across the nonlinear parameter $\alpha$ reveal a non-ideal parameter identifiability process. As an alternative, the *vOG* and *vEXP* models can be employed if more accuracy is required, as they lead to the identification of a clear minima and also provide an improved fit. They are more complex, involving two nonlinear parameters. Furthermore, the *vOG* model relies on the computation of eigenvalues, which is more computationally expensive.

Considering this study’s findings, the *vEXP* model provides a good balance between model complexity and data fit. The use of the parameter scaling norm was essential in ensuring a good overall model fit. Nevertheless, note that this norm was employed in order to overcome strain softening effect. Ideally, this effect would be mitigated by preconditioning the samples at the highest strain applied, but here, due to the very large strains, is avoided in order to avoid tissue damage to the samples during early testing.

### Extension and impact in vivo and in silico

The transportation industry is particularly concerned with the study of liver’s response to sudden large impacts, as it is the most exposed abdominal organ in frontal and side crashes. Having a model that is able to capture its behaviour at large deformations as well as rate-dependent effects enables the determination of injury tolerance limits and hence can guide the design of seatbelts, airbags, children car seats, etc. Although the models designed here are more appropriate for phenomena occurring repetitively, they can provide a starting point or complement the investigation of sudden high impacts experienced in injury crashes.

In the medical research field, in silico simulations are used in order to test new devices, provide surgery training, predict diagnosis and treatment response, etc. These simulations need to be able to gauge the nonlinear viscoelastic tissue response to a range of mechanical stimuli, e.g. cutting, puncturing, pressing. The models discussed here are suitable for in silico work, with the *vEXP* model performing better than the *vMR*$^*$ model and being computationally cheaper than *vOG*.

A different possible application of the models discussed is in the medical elastography area, which provides non-invasive in vivo measurements of an organ’s stiffness in order to assess disease severity. These measurements rely on assumptions about an underlying biomechanical model. In general, simple models are considered (e.g. Neo-Hookean), but frequency-dependent nonlinear viscoelastic models, like the ones presented here, could lead to a better identification and staging of pathology. With the liver being one of the most imaged organs using elastography, the 3D liver models proposed here could be translated and investigated in the context of small amplitude shear waves probing the liver at various deformation states imposed by the respiratory motion (Kang et al. 2012).

### Study limitations

The aim of this paper is to define a frequency-dependent nonlinear viscoelastic law that is able to characterise the liver tissue under a range of combined deformations and frequencies. The results presented advocate for the suitability of all three models employed, with *vEXP* in particular, to capture the liver behaviour, yet some limitations remain to be addressed by further research.

One of the main difficulties in this study was to overcome strain softening effect encountered, which was emphasised by the testing procedure. In a study performed by Cheng et al. (2009) on spinal cord, it was shown that the response of tissue loaded to 2% uniaxial strain differs significantly if the tissue is preconditioned to a maximum of 2% or 5% strain. Nonetheless, the tissue behaviour that was loaded up to 2% or 5% did not differ if all samples were preconditioned at 5% strain. This indicated that the strain softening phenomena can be addressed by preconditioning at the highest strain used in the testing protocol. In our study, given that large deformations were investigated, the preconditioning protocol had to be applied sequentially with the measurements. That is, at each deformation level, eight oscillations were carried out before acquiring the data, which was enough in order to obtain a steady response in the data, as shown in the Lissajous-type curves in Fig. 12. However, the tissue samples were not strained to the maximum deformation level before starting the data acquisition, as that could have damaged the samples’ microstructure and led to non-representative measurements at low strains. In order to address this effect, an initial preconditioning would need to be done at the highest deformation level, as suggested by Cheng et al. (2009), or measurements would need to be acquired in reverse, from the highest to the lowest strain. This would also require determination of the maximal deformation allowed avoiding material damage. An alternative approach in dealing with the strain softening would be to devise models that are able to capture this phenomenon. However, such models are usually heuristic (e.g. Perepelyuk et al. 2016), and their use is thus limited. Hence, the best approach in mitigating the strain softening remains preconditioning to the highest strain level applied in the testing protocol, despite that this level might be variable across studies.

A limitation of the *vOG* and *vEXP* models, compared to *vMR*$^*$, is the absence of a purely elastic term. Over an infinite time, the viscoelastic term modelled using the fractional-order derivative $D_t^\alpha$ would approach 0. Nonetheless, the time required for this decay is very large, as the normal force component in uniaxial compression relaxes proportionally to the power law $F \propto F_{\mathrm{max}} t^{-\alpha }$, where *F* is the force at time *t* and $F_{\mathrm{max}}$ is the maximum force reached due to deformation. For a fractional order similar to the one identified in this study, i.e. $\alpha =0.2$, the time required for the force to decay to 1% of the maximum value is $10^{10}$ s ($\approx$ 317 years). This is an impractical time duration for both experiments and simulations. Thus, despite the eventual decay of the force, here it was considered that a single viscoelastic term is appropriate for modelling the data. Adding a purely elastic term ${\varvec{S}}_e$ to the *vOG* and *vEXP* models was also considered, as exemplified in “Appendix 1”; however, this leads to parameter coupling. A broader frequency interval may be beneficial in identifying two separate elastic and viscoelastic terms, but for the data available in this study there was a trade-off between adding a purely elastic term and identifying a fractional order $\alpha$.

One of the assumptions made in the modelling process is the idealised compression employed. In the experiments, however, the samples bulged under compression. Likely, the viscoelastic model response would change when considering a non-idealised compression. Investigations in simulated 3D models indicate that there is a torque amplitude difference between the idealised and non-idealised compression. However, this would have an impact only on the linear parameters of the models, which would need to be upscaled. The normal force, nonetheless, would be the most affected, as simulations show a qualitative difference between ideal and non-ideal compression. “Appendix 2” presents a detailed in silico comparison of normal forces and torque between ideal and non-ideal compression.

The liver samples used in this study were fixed in the rheometer using serrated plates. To ensure sample gripping, an initial compressive strain and force had to be applied before running the experiments, and hence, the total compressive force at 1, 10 and 20% compression strain was 0.07, 0.2 and 0.7N, respectively. Despite the initial preload, other fixing methods like glue could have led to changes in tissue properties (Nicolle et al. 2010) and artificial changes in the samples’ height.

Although the range of frequencies investigated here is small (0.5–2Hz), the models presented in this study are applicable for other frequencies as well. The fractional order $\alpha$ is related to the frequency response, and the value identified in this study ($\alpha =0.2$) is in good agreement with literature findings (Liu and Bilston 2000; Jordan et al. 2011; Sinkus et al. 2018; Asbach et al. 2008). Thus, by choosing the nonlinear parameters presented in Fig. 9 and the appropriate linear parameters corresponding to a certain deformation level (Fig. 14), the models can be used for predicting phenomena occurring repetitively, at various frequencies. (e.g. for MRE purposes). However, when a sudden force is experienced (e.g. crash injury), the response is hard to predict, and hence, the use of these models in an area like transportation industry is limited.

The current model presented considers liver tissue as viscoelastic, neglecting porous media effects due to the vasculature of the liver. Preparation of tested samples was selected with care to avoid large vessels that may bias measurements; however, smaller vessels were present throughout the test samples. While the presence of vasculature can exhibit behaviour similar to viscoelastic phenomena, studies neglecting viscoelastic response in cardiac tissue, arterial wall and articular cartilage (at strain rate $>0.0001$s$^{-1}$) have not been able to replicate experimentally observed hysteresis or relaxation (Yang and Taber 1991; Zakerzadeh et al. 2016; DiSilvestro et al. 2001). Further, as the constituents of the liver have been shown to exhibit viscoelastic material response (e.g. hepatic cells are viscoelastic (Zhang et al. 2002; Yang et al. 2019), extracellular collagen needs to exhibit viscoelasticity in order to ensure accurate hepatic cell behaviour (Mattei et al. 2018) and isolated collagen fibrils exhibit viscoelasticity (Shen et al. 2011)), we believe the observed behaviour stems from viscoelasticity within the material. Further studies considering poro-viscoelasticity (particularly at the whole-organ level) would provide an interesting avenue for further investigations.

## Conclusions

This paper investigates the suitability of three models—*vMR*$^*$, *vOG* and *vEXP*—to capture the nonlinear viscoelastic liver response at a range of large deformations. Their performance is gauged using three norms—the $L_2$ norm, a point-wise scaling norm, which ensures even contributions from each data point, and a novel parameter scaling norm, which allows for flexibility in determining the linear parameters. This norm was introduced in order to overcome the strain softening effect, and its implementation leads to significant improvements in fitting the models to the data, with the *vOG* and *vEXP* models being able to better capture the nonlinear particularities. Among the forms presented here, the *vEXP* model is able to recreate the data trends, while identifying a clear minima across the fractional order $\alpha$. Furthermore, it relies on strain metrics that are cheap to compute, making it advantageous to use.

## Figures and Tables

**Fig. 15:**
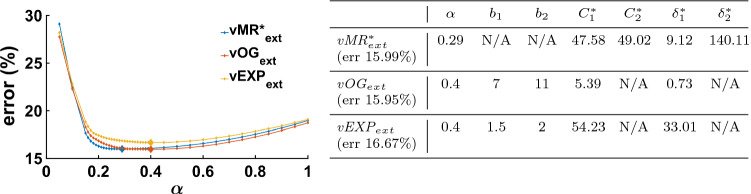
Minimum model error (*vMR*$^*_{\mathrm{ext}}$, *vOG*$_{\mathrm{ext}}$ and *vEXP*$_{\mathrm{ext}}$) for the parameter scaling norm (Eq. 30), obtained for the tests altogether, for fractional-order $\alpha$ values between 0.05 and 1. The minimal error across $\alpha$ is enhanced by the larger-sized marker, obtained for the parameters presented in the corresponding tables

**Fig. 16:**
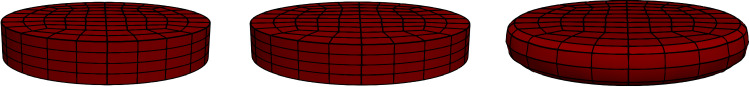
Simulations of liver sample compression; (left) liver in undeformed state; (middle) liver under 10% ideal compression; (right) liver under 10% non-ideal compression

**Fig. 17:**
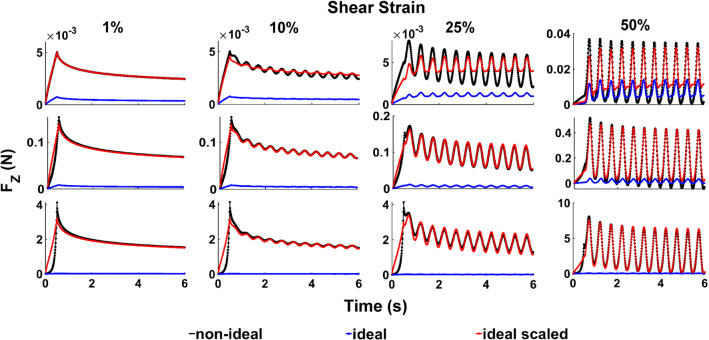
Normal force simulations during ideal and non-ideal compression. The simulations were carried out at CS 1% (top row), 10% (second row) and 20% (third row), and shear strain 1% (first column), 10% (second column), 25% (third column) and 50% (fourth column). The black curves show the non-ideal compression, the blue curves show the ideal compression, and the red curves show the scaled ideal compression, using the factors presented in Table 3

**Fig. 18:**
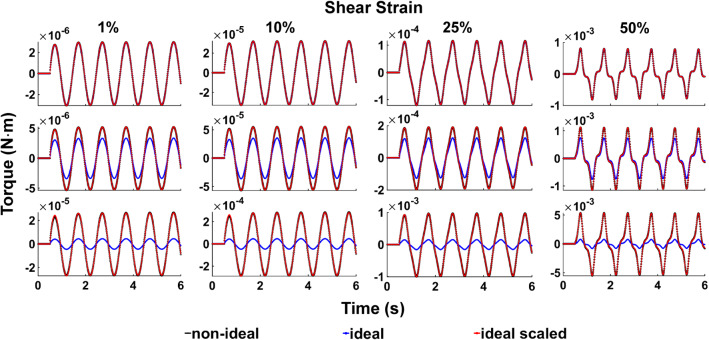
Torque simulations during ideal and non-ideal compression. The simulations were carried out at CS 1% (top row), 10% (second row) and 20% (third row), and shear strain 1% (first column), 10% (second column), 25% (third column) and 50% (fourth column). The black curves show the non-ideal compression, the blue curves show the ideal compression, and the red curves show the scaled ideal compression, using the factors presented in Table 2

**Fig. 19:**
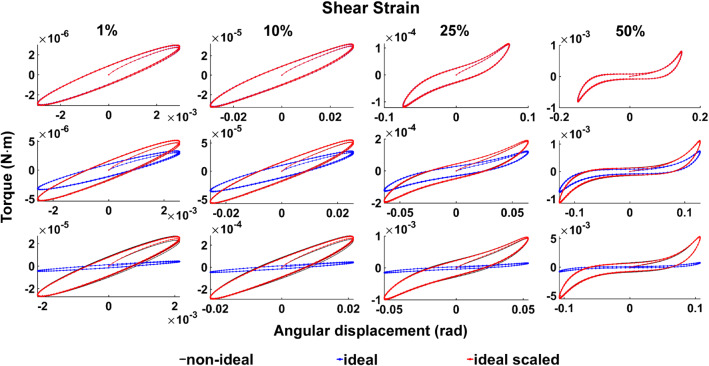
Lissajous curves of torque simulations during ideal and non-ideal compression. The simulations were carried out at CS 1% (top row), 10% (second row) and 20% (third row), and shear strain 1% (first column), 10% (second column), 25% (third column) and 50% (fourth column). The black curves show the non-ideal compression, the blue curves show the ideal compression, and the red curves show the scaled ideal compression, using the factors presented in Table 2

## Appendix 1: Extended models

The models presented in this study may be extended to include more components. This could allow for more flexibility in the modelling process. For example, consider the extended version of the *vMR*$^*$ model (*vMR*$^*_{\mathrm{ext}}$) to be

31 $$\begin{aligned} {\varvec{S}} = C_1 {\varvec{S}}_{\mathrm{e}}^1+C_2 {\varvec{S}}_{\mathrm{e}}^2+ \delta _1 D_t^ \alpha ( {\varvec{S}}_{\mathrm{e}}^1)+ \delta _2 D_t^ \alpha ({\varvec{S}}_{\mathrm{e}}^2 )+ {\varvec{S}}_{\mathrm{p}}, \end{aligned}$$

where $S_{\mathrm{e}}^{1,2}$ are defined as in the set of Eqs. 14. Similarly, the extended *vOG* model (*vOG*$_{\mathrm{ext}}$) would be characterised as

32 $$\begin{aligned} {\varvec{S}} = C_1 {\varvec{S}}_e^{b_1} + \delta _1 D_t^ \alpha ({\varvec{S}}_e^{b_2} )+ {\varvec{S}}_{\mathrm{p}}, \end{aligned}$$

with $S_{\mathrm{e}}^{b}$ given by Eq. 16. Lastly, the extended *vEXP* model (*vEXP*$_{\mathrm{ext}}$) would be defined as

33 $$\begin{aligned} {\varvec{S}} = C_1 {\text {Dev}} [ {\varvec{S}}_e^{b_1}] + \delta _1 {\text {Dev}} [D_t^\alpha {\varvec{S}}_e^{b_2}] + {\varvec{S}}_{\mathrm{p}}, \end{aligned}$$

where $S_{\mathrm{e}}^{b}$ is characterised by Eq. 18. For all three extended models, the material parameters *C* and $\delta$ are linearly scaling the elastic and viscoelastic contributions, respectively.

### Appendix 1.1: Results employing the extended models

Employing the extended models with the parameter scaling norm leads to smaller errors compared to the initial models. Figure 15 summarises the minimum errors and best parameter set for each model. The *vMR*$^*_{\mathrm{ext}}$ model leads to a better fit ($\approx$ 4% error improvement) compared to *vMR*$^*$. For the other two extended models, the error improvement is less than $\approx$1%. However, in Fig. 15 it can be seen that the minima are shallow, whereas when using the initial models *vOG* and *vEXP* (Fig. 9), the minima are more clearly identified. This is because the *vOG*$_{\mathrm{ext}}$ and *vEXP*$_{\mathrm{ext}}$ models offer more flexibility in fitting the data, by adding a purely elastic component. This becomes dominant, while the viscoelastic part is used for fine-tuning of the nonlinear behaviour, which is why the error curve is shallow across $\alpha$ values. Given the data range used in this study, the extended models do not lead to a clear parameter identifiability. Nevertheless, they might be suitable in modelling more complex experimental data or different materials.

## Appendix 2: Ideal and non-ideal compression

The modelling part of this study assumes ideal compression conditions, where the cylindrical samples can slide under compression and maintain a cylindrical shape. However, due to the nature of the rheological experiment, which employs serrated plates, the top and bottom faces of the samples were prevented from sliding, hence leading to non-ideal compression and a barrelling effect. This effect has been previously investigated in the literature (Mendis et al. 1995; Miller 2005; Wu et al. 2004; Spilker et al. 1990; Roan and Vemaganti 2007), and it was shown that the compressive force was significantly higher when a no-slip boundary was in place, leading to barrelling. This was more pronounced for samples where height/diameter ratio is small, as in the current study. In order to address discrepancies that might arise, here, we investigate the differences between ideal and non-ideal compression in an in silico setting. For this, ${\mathcal {C}}$**heart** (Lee et al. 2016) was used to solve the mechanical problem posed in Sect. 2.2.3.

In order to replicate the experiment as accurately as possible, a cylindrical sample of radius $R=10$ mm and height $H=3$ mm was compressed by 1, 10 and 20% and sheared up to 1, 10, 25 and 50% level, at a frequency of 1 Hz. The exponential model (Eq. 18) was employed, with the linear scaling $\delta =130$, the nonlinear parameter $b=1.5$ and the fractional power $\alpha =0.2$. The uniform compression was prescribed at the top and bottom surfaces as

34 $$\begin{aligned} \varvec{u} (t)= \left[ \begin{array}{c} \dfrac{X_1 }{\sqrt{\lambda (t)}} \cos (\psi (t) X_3) -\dfrac{ X_2 }{\sqrt{\lambda (t)}} \sin (\psi (t) X_3) - X_1 \\ \dfrac{X_1}{\sqrt{\lambda (t)}} \sin (\psi (t) X_3) + \dfrac{ X_2 }{\sqrt{\lambda (t)}} \cos (\psi (t) X_3) - X_2 \\ X_3 \left( \lambda (t)-1 \right) \\ \end{array} \right] , \end{aligned}$$

while the non-ideal compression was prescribed as

35 $$\begin{aligned} \varvec{u} (t)= \left[ \begin{array}{c} {X_1 }\cos (\psi (t) X_3) -{ X_2 }\sin (\psi (t) X_3) - X_1 \\ {X_1}\sin (\psi (t) X_3) + { X_2 } \cos (\psi (t) X_3) - X_2 \\ X_3 \left( \lambda (t)-1 \right) \\ \end{array} \right] . \end{aligned}$$

Note that the other boundaries were left unrestricted. Here, $\lambda$ is the compression and $\psi X_3$ is the shear angle, where

36 $$\begin{aligned} \psi (t)= \frac{\gamma \lambda ^{3/2}(t) \sin (2\pi f \hat{t}) }{\sqrt{X_1^2 + X_2^2}}. \end{aligned}$$

The compression was set to take place linearly over the course of 0.5 s, i.e. $\lambda (t) = 1 - {\text {CS}}\min (t,0.5)$, and the angular displacement was applied after the desired compression level was reached, i.e. $\hat{t} = \max (0,t-0.5)$.

Note that, while ideal compression was assumed in order to ensure rapid model evaluation, correction to non-ideal compression could be carried out for each case individually. However, the non-ideal model is time-consuming and costly, especially when analysing different model forms over a broad parameter space.

### Appendix 2.1: Results comparing ideal and non-ideal compression

The liver under ideal and non-ideal 10% compression is illustrated in Fig. 16. It can be seen that, under non-ideal compression, the sample is barrelling. Simulations of the normal force on the top surface and torque are presented in Figs. 17, 18 and 19.

The normal force simulations show the most striking differences between the two compression types, which is consistent with literature findings. In order to facilitate the comparison of the force between ideal and non-ideal compression, the force corresponding to the ideal case was upscaled by the parameters presented in Table 3. These were obtained by ensuring the best point-wise fit between the two cases, with the errors being reported for each test. It can be seen that for small compression and shear strains (1%), an almost perfect match can be recovered. The factor used in this case (6.5821) matches the correction indicated by Gent (2012) for short cylindrical samples (i.e. 6.5556, corresponding to the specific sample size considered here). The correction proposed by Gent stands for small strains, and hence, it can be seen that for the larger strains utilised in this study, the parameters are different and the match deteriorates.

Despite the discrepancy in the normal force, the ideal compression consideration does not invalidate this study. In Figs.  18 and 19, it can be seen that, although the torque amplitude differs between ideal and non-ideal compression, the trends are preserved and a good match can be obtained by upscaling the ideal compression case by the factors presented in Table 2. For 1% compression strain, the factor is almost unity, which is expected since very little barrelling is experienced under 1% compression. The errors when matching the two cases is generally very small. For 20% compression, the errors can reach 3%, but this is likely due to the first oscillatory cycle, as the remaining cycles look better matched.

Although there is a scale factor that needs to be applied to the torque simulations between ideal and non-ideal compression, this only affects the identification of the linear parameters presented in Figs. 9 and 13. Since the parameter scaling norm (Eq. 30) allows for the linear parameters to be adjusted according to each test, the ideal compression assumption does not impact the model fit to the data, but it introduces additional uncertainty on the linear parameter $\delta$. Note that, as the torque scaling factor is approximately constant across shear strain (for compression strain 1% and 10%) or increases (for the CS 20% group), consideration of non-ideal compression would not have resolved the shear strain softening phenomena observed in the study, as shown in Fig. 14. Overall, it can be concluded that the exponential model (18) with the nonlinear parameters presented in Fig. 9 and the linear parameters presented in Fig. 14 is suitable for capturing the constitutive behaviour of liver tissue.

**Table 3:** Multiplying factors between ideal and non-ideal torque and normal force measurements

Force	SS 1%	SS 10%	SS 25%	SS 50%
CS 1%	6.5821	6.0163	4.0603	2.2828
	0.2199%	6.4098%	21.9181%	29.6016%
CS 10%	15.5741	15.3684	14.2890	11.6911
	8.6189%	8.1965%	6.8576%	13.1985%
CS 20%	122.6414	121.2497	113.4665	93.5268
	16.0961%	15.6841%	14.1744%	8.3436%
